# A Trans-Amazonian Screening of mtDNA Reveals Deep Intraspecific Divergence in Forest Birds and Suggests a Vast Underestimation of Species Diversity

**DOI:** 10.1371/journal.pone.0040541

**Published:** 2012-07-17

**Authors:** Borja Milá, Erika S. Tavares, Alberto Muñoz Saldaña, Jordan Karubian, Thomas B. Smith, Allan J. Baker

**Affiliations:** 1 National Museum of Natural Sciences, Spanish Research Council (CSIC), Madrid, Spain; 2 Center for Tropical Research, Institute for the Environment, University of California Los Angeles, Los Angeles, California, United States of America; 3 Royal Ontario Museum, Toronto, Canada; 4 Department of Ecology and Evolutionary Biology, Tulane University, New Orleans, Louisiana, United States of America; 5 Department of Ecology and Evolutionary Biology, University of California Los Angeles, Los Angeles, California, United States of America; University of Guelph, Canada

## Abstract

The Amazonian avifauna remains severely understudied relative to that of the temperate zone, and its species richness is thought to be underestimated by current taxonomy. Recent molecular systematic studies using mtDNA sequence reveal that traditionally accepted species-level taxa often conceal genetically divergent subspecific lineages found to represent new species upon close taxonomic scrutiny, suggesting that intraspecific mtDNA variation could be useful in species discovery. Surveys of mtDNA variation in Holarctic species have revealed patterns of variation that are largely congruent with species boundaries. However, little information exists on intraspecific divergence in most Amazonian species. Here we screen intraspecific mtDNA genetic variation in 41 Amazonian forest understory species belonging to 36 genera and 17 families in 6 orders, using 758 individual samples from Ecuador and French Guiana. For 13 of these species, we also analyzed trans-Andean populations from the Ecuadorian Chocó. A consistent pattern of deep intraspecific divergence among trans-Amazonian haplogroups was found for 33 of the 41 taxa, and genetic differentiation and genetic diversity among them was highly variable, suggesting a complex range of evolutionary histories. Mean sequence divergence within families was the same as that found in North American birds (13%), yet mean intraspecific divergence in Neotropical species was an order of magnitude larger (2.13% vs. 0.23%), with mean distance between intraspecific lineages reaching 3.56%. We found no clear relationship between genetic distances and differentiation in plumage color. Our results identify numerous genetically and phenotypically divergent lineages which may result in new species-level designations upon closer taxonomic scrutiny and thorough sampling, although lineages in the tropical region could be older than those in the temperate zone without necessarily representing separate species. In-depth phylogeographic surveys are urgently needed to avoid underestimating tropical diversity, and the use of mtDNA markers can be instrumental in identifying and prioritizing taxa for species discovery.

## Introduction

Species richness in highly diverse and relatively understudied tropical regions is severely underestimated. Recent molecular systematic studies on tropical taxa have revealed that traditionally accepted species-level taxa often conceal genetically divergent subspecific lineages found to represent true biological species upon close taxonomic scrutiny [1]–[4]. A more accurate estimate of tropical diversity requires establishing species limits in numerous taxa for which little information on geographic variation is often available. In birds, robust taxonomic assessment of taxa for establishing species limits requires extensive field sampling across often large geographic ranges and labor-intensive analysis of both molecular and phenotypic datasets. In Amazonian birds, the challenge is particularly daunting, as evidenced by the fact that adequate phylogeographic studies documenting patterns of intraspecific genetic structure are available for about 1% of the more than 1000 species in the region.

Given the magnitude of the task at hand, time- and cost-effective techniques are needed to identify potential taxa and lineages of interest, which can then be subjected to in-depth taxonomic study. The use of mitochondrial DNA markers to describe patterns of intraspecific genetic structure and reveal divergent lineages provides a relatively efficient approach to detecting potential new species [2], [5], [6]. Although molecular data are not in themselves sufficient for species designation [7], they provide valuable information on patterns of lineage divergence and gene flow among populations that, when combined with data on phenotypic traits (e.g., plumage coloration, song and behavior) can be instrumental in designating species-level taxa [8], [9].

Variation in mtDNA coding genes such as cytochrome *c* oxidase I (COI), has shown reasonably good congruence with species boundaries in birds of North America [10], [11], the Palearctic [12], Argentina [13], [14], South America [15] and the Korean Peninsula [16], revealing small intraspecific differences (<1% divergence) relative to differences among species. An emerging pattern in these regional assessments of COI variation is one of higher intraspecific divergence and more marked phylogeographic structure in the tropics than in temperate areas, with a number of genetically isolated populations suggesting the existence of species not recognized by current taxonomy [15]. As one of the most diverse areas within the Neotropical region, the Amazon basin has received relatively little attention, although phylogeographic studies conducted to date in a limited number of Amazonian species [17]–[23] indicate that (1) intraspecific genetic distances are often larger than in the temperate zone, (2) lineage phylogeny often does not match current subspecific taxonomy, and (3) some intraspecific lineages are likely to represent new species given congruent levels of genetic and phenotypic divergence. This emerging pattern suggests a potentially serious underestimation of Amazonian avian richness [24], [25], and warrants increased research emphasis on intraspecific phylogeography across multiple taxa.

**Figure 1:**
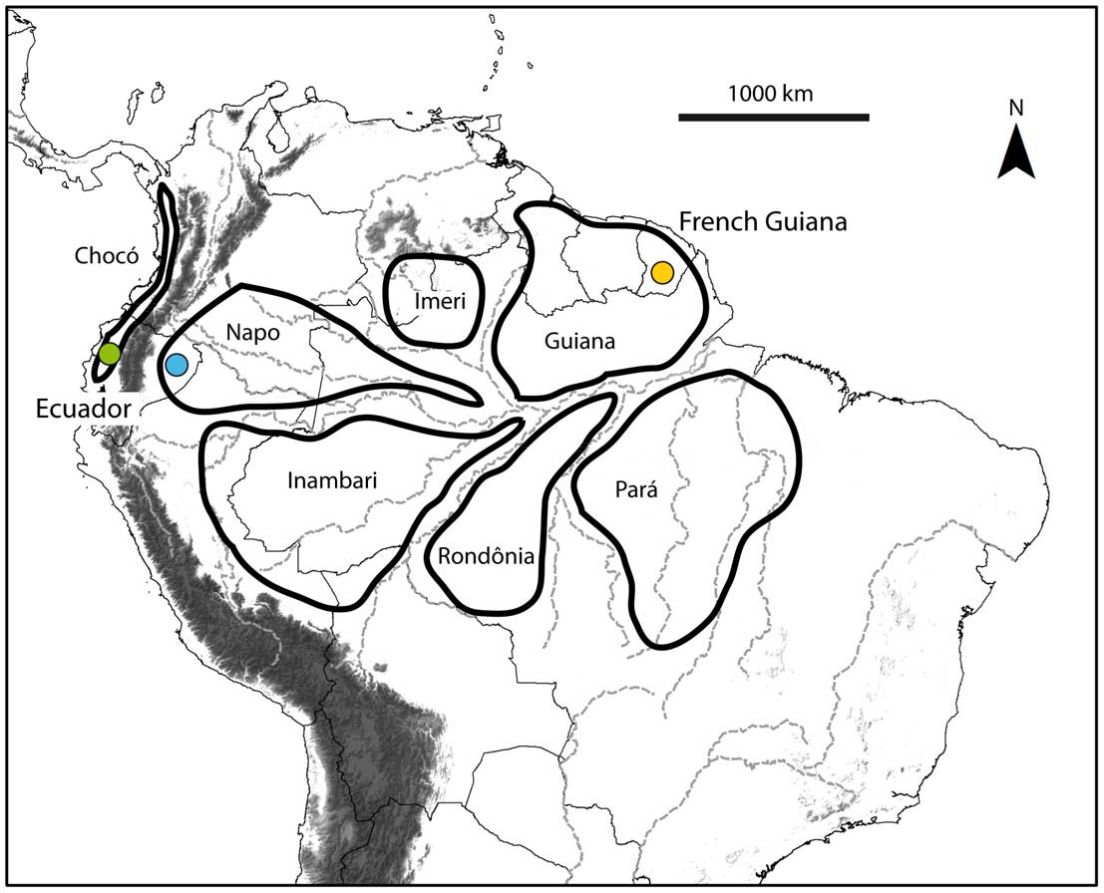
Map of northern South America showing the main areas of avian endemism according to Cracraft [**26**], and the three main sampling areas in the west-Ecuadorian Chocó (green), eastern Ecuador in western Amazonia (blue), and French Guiana in eastern Amazonia (yellow). Detailed locality data are provided in Table S1.

Here we screen patterns of variation in the cytochrome *c* oxidase I (COI) gene across 42 species of Amazonian forest birds by comparing sequences from individuals within and between two trans-Amazonian areas: the lowlands of eastern Ecuador in western Amazonia, and French Guiana in eastern Amazonia (Fig. 1). For some of the taxa, we also include sequences from the Chocó region of western Ecuador, which is separated from the Amazon basin by the Andes cordillera, to assess the relative contribution of trans-Andean differentiation to intraspecific variation in those species. These three sampling areas correspond to three of the eight main regions of avian endemism in tropical South America [26], and the large geographic distances involved relative to the species ranges ensure that observed patterns of divergence are likely to be relevant in the context of overall intraspecific variation. The 43 species included in the analysis belong to 6 orders and 17 families, 13 of them within the order Passeriformes (Table 1), and correspond mainly to *terra firme* lowland tropical forest specialists.

Our specific objectives are to (1) describe patterns of trans-Amazonian intraspecific genetic distances for a subset of forest bird species, for most of which no molecular data were previously available; (2) to compare patterns of intraspecific mtDNA divergence with those found in better-studied temperate zone species; (3) to assess whether patterns of intraspecific genetic divergence are generally associated with patterns of plumage color divergence; (4) to identify potentially polyphyletic taxa and divergent intraspecific lineages that may represent potential new species and thus deserve future in-depth taxonomic scrutiny; and (5) to evaluate the prospect of mtDNA gene sequences as markers for avian species discovery in the Neotropics.

**Table 1:** Study taxa, sample sizes, number of COI haplotypes found, and percent K2P genetic distances among populations corrected for intra-population polymorphism (D_xy_).

Family	Species	n (EE, WE, FG)	No. Haps.(EE, WE, FG)	%Dxy(EE - FG)	%Dxy(EE -WE)	%Dxy(WE - FG)	Mean Lineage Dist. (%)	Max. Lineage Dist. (%)	Min. Lineage Dist. (%)	Mean Intrasp. Dist. (%)
**Columbidae**	*Geotrygon montana*	5, 1, 2	3, 1, 2	0.10	0.08	0.07	0.10	0.28	0.00	0.11
**Trochilidae**	*Phaethornis bourcieri*	7, 0, 7	5, 0, 2	4.19	–	–	4.19	4.56	3.92	2.35
	*Campylopterus largipennis*	4, 0, 5	3, 0, 4	4.12	–	–	4.12	4.37	3.74	2.41
	*Thalurania furcata/fannyi*	13, 6, 10	9, 2, 4	3.64	2.79	3.69	3.44	4.48	2.49	2.39/0.74
**Trogonidae**	*Trogon rufus*	2, 4, 1	2, 1, 2	2.53	5.01	5.57	3.63	5.28	2.38	3.39
**Alcedinidae**	*Chloroceryle aenea*	2, 0, 1	2, 0, 1	0.14	–	–	0.14	0.14	0.14	0.14
**Momotidae**	*Momotus momota*	1, 0, 1	1, 0, 1	1.50	–	–	1.50	1.50	1.50	1.00
**Galbulidae**	*Galbula albirostris*	3, 0, 5	1, 0, 2	3.87	–	–	3.87	3.90	3.75	2.10
**Furnariidae**	*Philydor erythrocercum*	2, 0, 6	2, 0, 5	5.32	–	–	5.32	5.65	5.09	2.41
	*Automolus ochrolaemus*	1, 3, 3	1, 3, 3	7.45	2.24	6.66	6.19	7.50	2.09	4.49
	*Xenops minutus*	9, 10, 2	2, 5, 2	5.37	5.21	6.49	5.50	6.99	5.04	3.41
	*Sclerurus mexicanus*	6, 3, 1	1, 1, 1	2.28	6.20	5.86	5.33	6.20	2.28	3.21
**Dendrocolaptidae**	*Dendrocincla fuliginosa*	7, 13, 4	3, 4, 2	5.04	2.56	5.43	3.92	6.13	2.34	2.63
	*Glyphorynchus spirurus*	20, 15, 22	6, 3, 7	4.97	4.37	4.65	4.76	5.55	4.13	3.29
**Thamnophilidae**	*Thamnomanes ardesiacus*	27, 0, 21	6, 0, 9	1.65	–	–	1.65	2.03	0.14	0.93
	*Thamnomanes caesius*	7, 0, 5	3, 0, 1	2.79	–	–	2.79	2.88	2.73	1.51
	*Myrmotherula axillaris*	20, 9, 11	7, 1, 3	0.33	0.54	0.74	0.53	0.85	0.28	0.40
	*Myrmotherula longipennis*	6, 0, 11	4, 0, 3	5.03	–	–	5.03	5.36	4.73	2.41
	*Myrmotherula menetriesii*	6, 0, 4	5, 0, 4	1.54	–	–	1.54	1.83	1.26	0.95
	*Hypocnemis cantator*	3, 0, 6	3, 0, 2	3.76	–	–	3.76	4.39	2.74	2.03
	*Hylophylax naevius*	20, 0, 11	10, 0, 6	1.02	–	–	1.02	1.30	0.72	0.59
	*Hylophylax poecilinotus*	26, 0, 19	13, 0, 9	7.01	–	–	7.01	7.65	6.51	3.66
	*Pithys albifrons*	24, 0, 21	2, 0, 11	0.77	–	–	0.77	1.86	0.57	0.46
**Formicariidae**	*Formicarius analis*	5, 0, 1	3, 0, 1	1.45	–	–	1.45	2.03	1.01	1.33
	*Formicarius colma*	6, 0, 9	4, 0, 6	3.70	–	–	3.70	3.90	3.61	0.91
**Conopophagidae**	*Conopophaga aurita*	6, 0, 2	2, 0, 1	4.02	–	–	4.02	4.15	4.00	1.75
**Tyrannidae**	*Mionectes oleagineus/macconnelli*	30, 0, 17	4, 0, 7	2.70	–	–	2.70	3.80	2.46	0.14/0.17
	*Corythopis torquatus*	1, 0, 7	1, 0, 3	1.30	–	–	1.30	2.06	1.17	0.57
	*Rhynchocyclus olivaceus*	2, 0, 4	2, 0, 3	8.58	–	–	8.58	8.75	8.40	4.68
	*Platyrinchus coronatus*	7, 7, 10	1, 5, 3	4.99	7.71	5.78	6.07	7.95	4.92	4.20
	*Myiobius barbatus*	4, 0, 6	3, 0, 2	2.61	–	–	2.61	3.06	2.46	1.54
**Pipridae**	*Pipra erythrocephala*	17, 5, 6	5, 2, 6	0.33	2.00	2.15	1.24	2.50	0.00	0.78
	*Dixiphia pipra*	19, 0, 26	7, 0, 11	3.67	–	–	3.67	4.70	1.33	2.22
**Tityridae**	*Schiffornis turdina*	5, 5, 4	3, 4, 2	9.69	4.42	9.96	8.05	11.11	3.23	6.14
**Vireonidae**	*Hylophilus ochraceiceps*	6, 0, 2	5, 0, 2	6.77	–	–	6.77	7.08	6.45	3.08
**Turdidae**	*Turdus albicollis*	9, 0, 18	4, 0, 8	0.25	–	–	0.25	1.00	0.00	0.25
**Troglodytidae**	*Cyphorhinus arada*	2, 0, 5	1, 0, 3	6.81	–	–	6.81	6.97	6.65	3.30
	*Microcerculus bambla*	3, 0, 2	2, 0, 1	5.28	–	–	5.28	5.33	5.18	3.20
**Thraupidae**	*Tachyphonus surinamus*	5, 0, 9	5, 0, 4	1.82	–	–	1.82	1.92	1.64	0.98
	*Lanio fulvus*	2, 0, 3	3, 0, 1	1.59	–	–	1.59	1.85	1.13	1.11
**Cardinalidae**	*Cyanocompsa cyanoides*	9, 3, 3	3, 3, 2	1.45	8.02	7.47	5.21	8.36	1.33	3.19

Mean, maximum and minimum lineage distances are based on trans-Amazonian and trans-Andean haplogroups. Mean intraspecific distance is the average divergence among all individuals in the species. EE  =  Eastern Ecuador, WE  =  Western Ecuador, FG  =  French Guiana.

## Materials and Methods

Birds were captured in the field using mist-nets at various localities in Ecuador (between 1999 and 2004) and French Guiana (between 2007 and 2008) (Fig. 1, Table S1). Each individual captured was identified, photographed, and a blood sample was collected by venipuncture for genetic analysis. Species selected for the study were those whose Amazonian distribution included both Ecuador and French Guiana, and we included trans-Andean samples from western Ecuador when available. Forty species were sampled in both east Ecuador and French Guiana, and twelve of them were also sampled in the west-Ecuadorian Chocó. Three additional species with more restricted ranges were included in the analysis for comparative purposes: two sister species within the flycatcher genus *Mionectes* (*M. oleagineus* and *M. macconnelli*, the former widely distributed in Amazonia and the latter restricted to the Guianan Shield), and *Thalurania fannyi*, a hummingbird species restricted to the Chocó but closely related to *T. furcata*, one of the species used in the trans-Amazonian comparison. In total we obtained sequences from 758 individuals (Table 1, Table S1). We follow Restall et al. [27] for species taxonomy and nomenclature.

DNA was extracted from blood by membrane purification with 96-well glass fiber filtration plates (Acroprep 96 Filter Plate, 1.0 µm Glass, PALL Corp.) [28]. Cycle conditions were based on previous analyses [29]. A stable segment of ∼910 bp of the cytochrome oxidase I gene was amplified with primers LTyr (TGTAAAAAGGWCTACAGCCTAACGC, Oliver Haddrath, pers. com.), and COI907aH2 (GTRGCNGAYGTRAARTATGCTCG, [29]). Amplified products were purified by excising bands from the agarose gel and filtering each through a filter tip [30], then sequenced in both directions using an automated sequencer ABI 3730 (*Applied Biosystems*), according to the manufacturer’s suggested protocols. The primers used in the sequencing reaction were Ltyr and COI748Ht (TGGGARATAATTCCRAAGCCTGG, [29]), except for *Platyrinchus coronatus*, for which COI907aH2 was used as the reverse primer instead.

We aligned sequences using Sequencher 4.1.4 (Gene Codes) and Geneious 5.3.6 (Biomatters), and polymorphisms were checked visually for accuracy. Sequences have been deposited in GenBank, and original trace files are available in the BOLD project “Neotropical Birds” (www.barcodinglife.org). We calculated sequence divergence using a Kimura-two-parameter (K2P) model of sequence evolution and corrected distances across the Amazon (trans-Amazonian) and across the Andes (trans-Andean) for intra-population polymorphism using Arlequin 3.1 [31]. All samples within each of the three main regions (French Guiana, East Ecuador and West Ecuador) were grouped together to estimate intra-population polymorphism. To examine phylogenetic relationships among haplotypes we constructed haplotype networks for each species using the median-joining algorithm in the program Network 4.6.0 (Fluxus Technologies Inc.). Statistical significance of network branches was estimated by 1000 bootstrap replicates on neighbor-joining trees generated for each species in MEGA 5.0 [32].

We calculated genetic diversity indices and demographic history parameters for Amazonian species with sufficient sample sizes (n ≥6 in both Ecuador and French Guiana). We calculated haplotype and nucleotide diversity indices in DnaSP [33] and tested for rapid changes in population size indicating past population expansions using Fu’s test of neutrality [34] and calculated values of F_s_ in Arlequin 3.1 [31].

We estimated phenotypic differences among intraspecific lineages by scoring the degree of plumage color divergence in at least one of the sexes, using a four-code key and the following arbitrary criteria: (1) no apparent differences in plumage and no subspecific designations in current taxonomy; (2) slight but diagnosable differences in color shade, intensity or extension, but no difference in the color itself or in color patterning; (3) marked differences in color shade, intensity or extension, but no differences in the color itself or in color patterning; (4) marked divergence in color or patterning. To standardize scoring as much as possible, we restricted the sources of subspecific phenotypic information to the color plates and verbal descriptions in Restall et al. [27] and our own photographic vouchers. Summary descriptions of phenotypic differences on which scores were based are provided in Table S2 in the Supplementary Materials. To test for an association between genetic distances and phenotypic scores we used a model II simple linear regression with a major axis (MA) regression method as implemented in the *lmodel2* package in R 2.10.1 (R Development Core Team).

The plumage divergence scores used here represent a very coarse estimate of differentiation, and were produced with two main objectives in mind: (1) showing overall patterns of genetic-phenotypic association (or lack thereof), and (2) identifying potential candidate lineages for new species designation, with the understanding that detailed phenotypic (including plumage, song, and behavioral data) and molecular analyses will be necessary before final taxonomic decisions are made. We realize that for taxonomic purposes, proper analysis of variation in plumage color should be based on more quantitative measures of color (such as those provided by spectrophotometry) taken on study skins rather than qualitative assessments extracted from color plates, verbal descriptions and subspecific designations in Restall et al. [27], although the latter are of course the result of extensive examination of study skins. We are also aware of the fact that human perception likely underestimates color differences perceived by the avian eye [35], [36], yet because most avian alpha taxonomy has been based on qualitative, subjective assessments of differences in plumage color as perceived by human taxonomists, we feel the method is justified within the confines of the stated objectives.

## Results

### Patterns of Intraspecific Genetic Divergence

**Figure 2:**
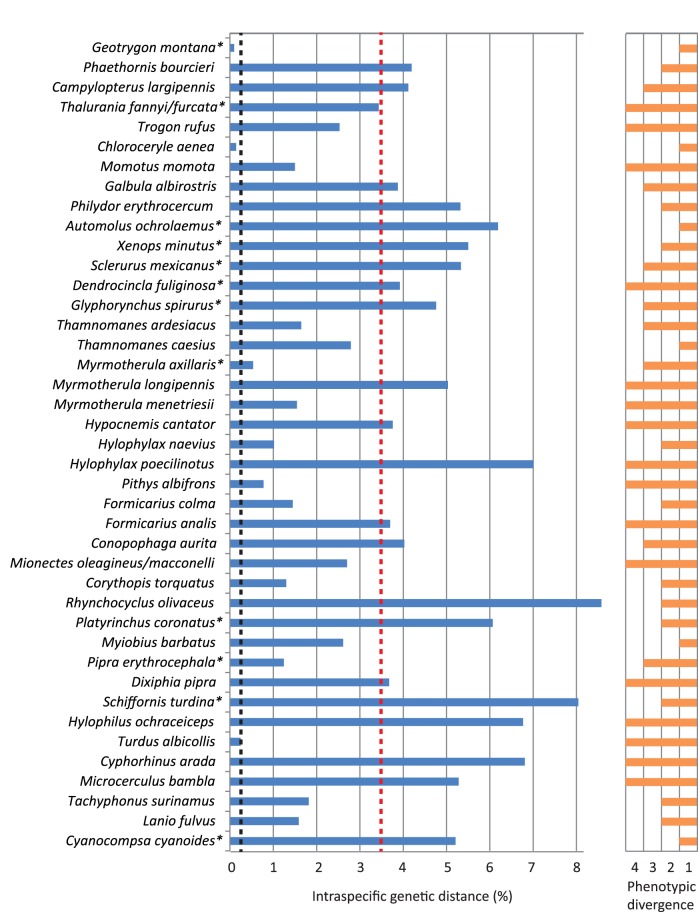
Genetic distances among intraspecific populations of 42 species of Neotropical birds across the Amazon basin and the Andes. Blue histograms correspond to total mean distances, calculated as percent K2P divergence corrected for intrapopulation polymorphism (see Table 2). Orange histograms represent values for phenotypic differentiation (plumage color divergence scored between 1 and 4, see Table S2). The red hatched line represents the average percent value for intraspecific lineage divergence for species in this study, and the black hatched line represents intraspecific divergence in temperate species from North America [11] and temperate and subtropical areas of Argentina [14].

**Figure 3:**
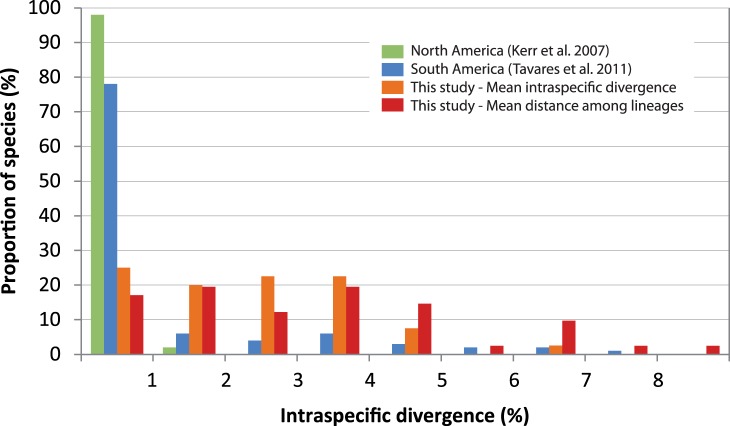
Intraspecific divergence in bird species of temperate and tropical areas. Green bars: percent mean intraspecific divergence in North American bird species examined by Kerr et al. (2007) [11]; Blue bars: percent mean intraspecific divergence found in South American species examined by Tavares et al. (2011) [15]; Red bars: percent mean distance among intraspecific lineages in 40 Neotropical species included in this study; Orange bars: percent mean intraspecific divergence in 40 Neotropical species included in this study. Mean intraspecific divergence is the average K2P distance among all sequences within a species, whereas mean distance among intraspecific lineages is the mean distance among divergent haplogroups across the Amazon and across the Andes (see Table 1). *Thalurania fannyi*, *Mionectes oleagineus* and *M. macconnelli* not included.

The majority of species used in the trans-Amazonian intraspecific comparisons in our sample (33 of 40, excluding *Thalurania fannyi* and the interspecific comparison in *Mionectes*), showed a consistent and clear separation between Ecuadorian and French Guianan haplogroups (Table 1, Fig. 2). Mean divergence in intraspecific COI lineages was 3.45% across the Amazon basin and 3.85% across the Andes, for a total mean value of intraspecific lineage divergence for our sample of 3.56%. The magnitude of the divergence was highly variable in different species, yet over 50% of them showed intraspecific lineage distances above 3%, much larger than the 0.23% mean intraspecific divergence found within species in North America and temperate regions of South America (0.24%) (Figs. 2, 3). Mean intraspecific divergence, which takes into account overall divergence among all sequences and not just the distance among divergent intraspecific lineages, was 2.13%, an order of magnitude larger than that found in temperate regions (Fig. 3). In temperate areas, mean intraspecific divergence below 1% was found in about 94% of species in North America and Argentina [11], [13], compared to only 25% of species in our Neotropical sample (Fig. 3). This percentage is also lower than that reported in a study on species from throughout South America (78%, [15]), although the study included many species from temperate areas and more limited intraspecific sampling.

**Figure 4:**
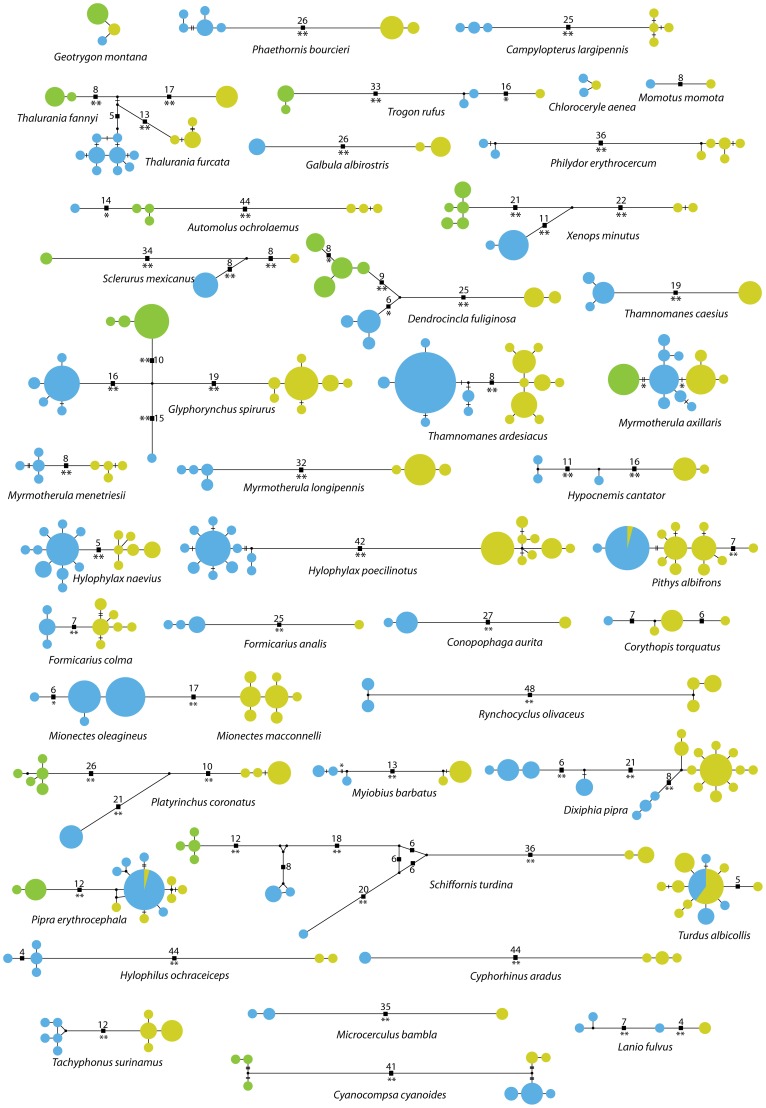
Median-joining haplotype networks of 43 Neotropical bird species. Circles represent individual COI haplotypes, their size is proportional to their frequency in the sample, and colors correspond to those used in Fig. 1. Branches among haplotypes correspond to one nucleotide change and hatch marks represent additional changes. Figures next to black squares along branches indicate the number of nucleotide changes when greater than 3. Asterisks indicate node support from a bootstrap analysis (1000 replicates) on a neighbor-joining tree (*: 90–96%, **: 97–100%). Species appear in phylogenetic order (see Fig. 2).

Within the main group of 33 species showing clear divergence across Amazonia, some also showed complex patterns of lineage divergence (Fig. 4). Indeed, some lineages were almost as divergent within eastern Ecuador and French Guiana as between the two areas across Amazonia (*Thalurania furcata, Glyphorynchus spirurus, Hypocnemis cantator, Dixiphia pipra, Schiffornis turdina,* and *Lanio fulvus*). In contrast, 5 species (*Geotrygon montana, Chloroceryle aenea, Myrmotherula axillaris, Hylophylax naevius* and *Cyanocompsa cyanoides*) showed limited divergence among haplotypes within both regions (<1%). Only 3 species (*Pithys albifrons, Pipra erythrocephala* and *Turdus albicollis*) showed no divergence across Amazonia and shared at least one haplotype among localities, although one of them (*Pithys albifrons*) showed marked differences in haplotype frequencies (Fig. 4).

The 12 species for which we had west-Ecuadorian Chocó samples in addition to Amazonian samples allowed us to obtain a better measure of intraspecific divergence and compare the relative magnitude of intraspecific distances across the Andes and across Amazonia. Five of the 12 species showed greater divergence across the Andes than across the Amazon (*Trogon rufus, Sclerurus mexicanus, Platyrinchus coronatus, Pipra erythrocephala* and *Cyanocompsa cyanoides*), 3 showed greater divergence across the Amazon than across the Andes (*Automolus ochrolaemus, Dendrocincla fuliginosa,* and *Schiffornis turdina*), and 4 of them showed approximately equal distance between the three haplogroups (*Geotrygon montana, Glyphorynchus spirurus, Myrmotherula axillaris,* and *Xenops minutus*) (Table 1, Fig. 4). In the hummingbird genus *Thalurania*, the distance between the trans-Andean species *Thalurania fannyi* and the east-Ecuadorian haplogroup of *T. furcata* was actually smaller than the distance between trans-Amazonian populations of *T. furcata*, making the latter paraphyletic.

### Genetic Diversity and Demographic History

For a subset of species with sufficient sample sizes (n ≥6 in both Amazonian regions), we calculated indices of genetic diversity and values of F_s_ to test for past events of rapid population growth. As with intraspecific genetic distances, patterns of genetic diversity and demographic change revealed high heterogeneity across species (Table 2), suggesting diverse demographic and evolutionary histories. Nucleotide diversity ranged from 0 in *Platyrinchus coronatus* to 0.266 in *Dixiphia pipra*, with higher values reflecting the presence of divergent intraspecific lineages within a population, and inter-population comparisons reaching differences above an order of magnitude in three species (*Pithys albifrons*, *Dixiphia pipra* and *Platyrinchus coronatus*). Haplotypic diversity was generally similar in trans-Amazonian comparisons, with marked differences detected only in *Pithys albifrons* and *Platyrinchus coronatus*. F_s_ values revealed marked differences in demographic history between trans-Amazonian populations of several species, with some showing evidence of past expansions only in western Amazonia (*Thalurania, Myrmotherula axillaris, Hyllophylax poecilinotus* and *H. naevius*) and some only in eastern Amazonia (*Glyphorynchus*, *Thamnomanes*, *Pithys*, *Dixiphia*, *Mionectes macconnelli* and *Turdus*). When both populations were combined, *Turdus albicollis* showed a significant signature of population growth (F_s_  =  −5.350, *P* = 0.003), suggesting a recent Amazon-wide population expansion.

**Table 2:** Genetic diversity and population expansion indices of trans-Amazonian populations.

Species	*π* (EE)	*π* (FG)	*h* (EE)	*h* (FG)	F_s_ (EE)	F_s_ (FG)
*Phaethornis bourcieri*	0,081	0,015	0,857	0,476	−1,262	0,589
*Thalurania furcata*	0,003	0,024	0,923	0,711	−5,094***	7,792
*Glyphorynchus spirurus*	0,006	0,001	0,574	0,645	1,612	−3,287**
*Thamnomanes ardesiacus*	0,066	0,078	0,453	0,838	−0,945	−3,652*
*Myrmotherula axillaris*	0,002	0,001	0,779	0,473	−2,318*	−0,659
*Myrmotherula longipennis*	0,040	0,014	0,867	0,473	−1,160	−0,659
*Hylophylax naevius*	0,080	0,115	0,832	0,836	−6,986***	−1,923
*Hylophylax poecilinotus*	0,032	0,043	0,819	0,836	−7,724***	−2,250
*Pithys albifrons*	0,004	0,123	0,083	0,871	−1,028	−3,888
*Dixiphia pipra*	0,266	0,029	0,836	0,748	5,717	−7,092***
*Pipra erythrocephala*	0,001	0,005	0,427	1,000	−1,533	−3,079*
*Platyrinchus coronatus*	0,000	0,001	0,000	0,378	0,000	0,300
*Mionectes oleagineus/macconnelli*	0,035	0,041	0,561	0,809	0,342	−3,247**
*Turdus albicollis*	0,073	0,123	0,750	0,850	−0,722	−2,550*

Included are 14 species of birds with sufficient sample sizes (n≥6). In the genus *Mionectes*, we compared *M. oleagineus* individuals from western Amazonia to the east-Amazonian species *M. macconnelli*. Indices shown include nucleotide diversity (π), haplotype diversity (*h*), and F_s_ values from Fu’s test of neutrality (see text). EE = Eastern Ecuador, FG = French Guiana.

* P<0.05.

** P<0.01.

*** P<0.001.

### Congruence between Genetic and Plumage Divergence

A cursory analysis of differentiation across Amazonia in overall plumage coloration revealed a wide range of patterns, with 6 species showing no apparent differences (value of 1), 11 species showing slight differences in shade or intensity (value of 2), 8 species showing marked differences in shade or intensity (value of 3), and 16 species showing marked differences in color and patterning (value of 4) (Table S2). The relationship between overall plumage divergence and genetic distance among trans-Amazonian haplogroups did not reveal a consistent pattern (Fig. 2) and the correlation between the two variables was not significant (r^2^ = 0.0083, n = 39, *P* = 0.290). Moreover, species with a phenotypic divergence value of 1 included both highly genetically divergent and closely related haplogroups (*Myiobius barbatus* and *Geotrygon montana*, respectively). The same was true for species with a plumage divergence value of 4, with species like *Hylophilus ochraceiceps* or *Cyphorhinus arada* showing high genetic divergence, yet some like *Pithys albifrons* showing very low divergence (Fig. 2).

## Discussion

### Intraspecific Divergence in Amazonian Birds

Our study reveals consistently large values of intraspecific divergence across the northern Amazon in 40 species of forest understory birds. The presence of clear genetic separation between trans-Amazonian samples in 33 out of 40 species studied suggests that this pattern might be widespread among Amazonian forest understory birds. The mean value of trans-Amazonian intraspecific lineage divergence in the COI gene was 3.45%, and including trans-Andean comparisons the total mean value of intraspecific lineage divergence for our sample was 3.56%. These values are about 15 times higher than those found for temperate areas of the world such as North America (0.23%) [11] and Korea (0.26%) [16], as well as temperate and sub-tropical areas of South America like Argentina (0.24%) [13], [14]. Our mean values of intraspecific lineage divergence are also higher than those found in a study of 561 species across South America [15], where over 75% of species had values of intraspecific divergence below 1%, although both temperate and tropical species were included in that study. Our results are instead consistent with the relatively few thorough species-specific phylogeographic studies conducted to date in Amazonian species, including *Pyrrhura picta-leucotis* (cyt *b*: 1.1–5.6%, 1.1–2.2% within *picta* group [21], *Glyphorynchus spirurus* (cyt *b* + ND2&3: 1.3–7.5%, [19]), *Xiphorhynchus elegans* (cyt *b*:1.6–1.9% [17], *Dendrocincla fuliginosa-merula* (cyt *b*: 0.5–6.8%, ND2: 0.11–9.0%, COI: 0.17–6.5% [23]), *Lepidothrix coronata* (cyt *b* + ND2&3: 1.5–4.3%, [18]), *Schiffornis turdina* (cyt *b* + COI + ND2: 0.8–9.6%, [20]), or *Phaeotlypis fulvicauda-rivularis* (cyt *b* + COI&II + ND2+ ATPase 6&8: 4.6–6.1%, [37]). Our results suggest that tropical intraspecific distances are larger than those found in other latitudes. Although previous surveys of COI variation in temperate areas included limited within-species sampling and thus might have underestimated intraspecific divergence, most thorough phylogeographic studies of temperate species are consistent with low levels of genetic structure and divergence [38]–[48].

A critical question with important implications for taxonomy, evolutionary biology, and conservation, is whether intraspecific genetic distances in the Neotropics are indeed larger than in the temperate zone, or whether this pattern is instead an artifact of incorrect species limits that underestimate species richness. Recent studies suggest that rates of phenotypic evolution of plumage coloration and song characteristics, both typically involved in avian reproductive isolation, are slower in tropical areas than in the temperate zone [49], [50], which is consistent with studies suggesting that speciation rates are faster in the latter than in the former [51], [52]. Thus slower rates of reproductive isolation towards the equator would predict the existence of relatively divergent lineages that have not necessarily speciated. However, it is widely accepted that a high proportion of tropical bird species remains undescribed relative to the temperate zone, and this taxonomic bias is likely to have a strong influence on our understanding of the processes underlying the latitudinal gradient in species richness, so the issue remains a matter of debate [53]–[55].

As phylogeographic and alpha taxonomic research progresses, it will become apparent whether patterns of both intra and inter-specific genetic distances are similar across temperate and tropical latitudes. Despite our limited sampling, mean divergence among taxonomic families in this study was 13%, very similar to the 12% found in a North American birds [10] and the 13% found for a worldwide sample [56]. Whether this apparent uniformity at the family level also applies to the species level will require further research. Those cases in which molecular and phenotypic data have been used to revise species limits in previously polytypic tropical species, intraspecific distances have dropped to temperate zone values. For example, when *Schistocichla* (*Percnostola) leucostigma* was divided into *S. leucostigma* and *S. saturata,* intraspecific distance was reduced from 9% to less than 1% (ATPase 6+ND2+ND3, Braun et al. [57]), and the taxonomic split of *Capito niger* into four species reduced mean intraspecific divergence from 6% to again less than 1% (cyt *b*+ COI, Armenta et al. [58]).

The consistent pattern of trans-Amazonian divergence in most species we examined suggests both a pattern of long-term co-distribution and a shared response to past environmental events, both central dictums of comparative phylogeography [59]. Identifying the factors driving trans-Amazonian divergence will require additional sampling across the region to determine the degree to which the geographic distribution of haplogroups shows discrete discontinuities at contact zones associated with known geographic barriers (such as prominent tributaries of the Amazon river, like the Negro or Branco rivers), or whether divergence is the product of clinal variation across geographic distance [60]. Investigating these patterns through range-wide phylogeographic studies, preferably involving the collection of voucher specimens, will be essential to shed light on both evolutionary process and the resulting patterns of species richness.

### Divergent Lineages and Species Discovery

Our screening of mtDNA variation revealed the presence of divergent lineages both across and sometimes within trans-Amazonian sampling areas. Marked patterns of both genetic and phenotypic intraspecific divergence in some taxa suggest that subspecific lineages are likely to represent new species not recognized by current taxonomic treatments. This is well illustrated by taxa like *Cyphorhinus arada, Microcerculus bambla,* and *Hylophilus ochraceiceps,* all showing congruent divergence in mtDNA and phenotype (Fig. 2 and 1). In taxa with continuous distributions across the region, establishing species limits that are consistent with this variation will require thorough geographic sampling to determine patterns of gene flow and introgression at contact zones among forms (e.g., *Cyphorhinus* and *Hylophilus*). In contrast, species delimitation should be easier in cases like *Microcerculus bambla*, where the geographic range of the differentiated lineages is markedly allopatric [27].

### Limitations and Caveats of Our mtDNA Screening Approach

Our screening of intraspecific divergence is based largely on two main localities, which is unlikely to be representative of true intraspecific variation in many of the species targeted. As shown by the subset of species with trans-Andean samples, which considerably increased mean values of intraspecific divergence, our assessment is likely conservative. Previous studies have shown that other major geographic barriers in the region such as the Amazon river and many of its tributaries, or the Cerrado biome separating the coastal Atlantic forest of Brazil from the main Amazonian forest mass, can also account for marked genetic divergence within species of understory birds [15], [61].

Sampling for this study was carried out using mist nets located on the floor of *terra firme* rainforest, and the sampling is thus biased towards understory specialists. Speciose groups such as canopy specialists and species restricted to seasonally flooded habitats with potentially higher dispersal capacity have shown different biogeographic patterns and lower intraspecific divergence than understory species [62], [63]. For example, genetic studies conducted on species associated with rivers and seasonally flooded forests such as *Xiphorhynchus kienerii* and *X. obsoletus* [64] and *Chrysomus icterocephalus* [65] have revealed very low levels of sequence divergence and widespread haplotype sharing, likely due to higher rates of gene flow along waterways.

Because of their overwhelming predominance in the rainforest understory, our sample is also biased towards passerines, and our conclusions may not apply to other avian orders. However, our data suggest that most of the variance in genetic divergence and diversity is observed within species and between closely related species rather than between higher taxonomic levels (genera, families or orders). In fact, variation in intraspecific divergence can be high even among closely related congeners, as demonstrated by the three species of *Myrmotherula* antwrens included in the study. Divergence within *M. axillaris* was minimal both across the Amazon (0.43% between east Ecuador and French Guiana) and across the Andes (0.53% between east and west Ecuador), in sharp contrast to a 5% trans-Amazonian divergence in *M. longipennis* and an intermediate 1.5% in *M. menetriesii*. This range of intra-genus divergence values has also been shown within non-passerine genera [21], [66].

Finally, the use of a single mtDNA coding gene to detect divergent lineages has important limitations. Due to the relatively slow mutation rate of mtDNA coding regions, recently diverged species may show incomplete lineage sorting in mtDNA [21], [39], and cases of mtDNA introgression through past hybridization may conceal true levels of evolutionary divergence [67], [68]. Mitochondrial markers will continue to be useful for recovering inter- and intraspecific phylogenies and detecting divergent lineages [69], yet more variable markers including biparentally inherited loci will often be necessary to fully reveal Neotropical genetic structure, and thus estimates from a single mtDNA coding gene likely constitute a conservative estimate.

### Implications for DNA Barcoding in Neotropical Birds

The universality of COI as a barcode for bird identification, with its many practical applications [9], [70], remains promising even in tropical areas. The utility of this gene to identify a good proportion of tropical bird species has been previously demonstrated [13]–[15], and today its universality seems to be mostly limited by incorrect species limits in many groups. The diversity and complexity of phylogeographic patterns found to date indicate that most currently recognized Amazonian species contain markedly divergent phylogroups that may represent new, separate species. As in-depth phylogeographic studies of Neotropical species contribute to the establishment of proper species limits and reveal general patterns of intraspecific divergence, we will be able to assess whether the high across-species variance in intraspecific distances is a true characteristic of Neotropical taxa, or instead is an artifact of uneven taxonomic coverage. Specifically, it will become apparent whether patterns of variation converge towards those found in temperate regions, or whether intraspecific lineages in the Neotropics are indeed older and therefore more structured. To the extent that COI variation reveals marked structure within current species, sequences from COI and other mtDNA genes will be useful in species identification (as DNA barcodes) even if current intraspecific clades are raised to the level of taxonomic species in the future.

### The Need for a Phylogeographic Approach to Species Discovery

The use of mtDNA markers has already been instrumental in taxonomic revisions of several Neotropical avian groups [21], [66], [71]–[84], and molecular work produced in just the last decade has had a major impact on taxonomy. However, most studies to date have focused on interspecific phylogenies based on incomplete or limited subspecific sampling that has often prevented definitive resolution of species limits. Phylogeographic studies that survey genetic variation across entire geographic ranges encompassing known patterns of phenotypic variation are still rare, yet are essential to properly describe species limits and richness.

Because plumage color is almost always under natural and/or sexual selection, numerous cases of rapid plumage divergence [39], [85], plumage convergence in divergent taxa [86], and cryptic speciation [87], [88] have been reported that can lead to taxonomic classifications that do not reflect the evolutionary history of taxa [89]. Therefore, combining data from neutrally evolving molecular markers with phenotypic traits is essential for establishing taxonomic species limits that are biologically meaningful [90], [91] and truly contribute to taxonomic progress [92].

The establishment of proper species limits in many taxa will not only better reflect patterns of diversity in the Amazon relative to other tropical regions and the temperate zone, but should also significantly improve attempts at understanding patterns of endemism [62], the evolution of range size [93], diversification hypotheses [94]–[97], speciation rates [52], [98], species distribution models [99] and conservation strategies [100]–[102].

## Supporting Information

Table S1 **Specimens sampled per species and per locality.** Species are listed in alphabetical order.
(PDF)

Table S2 **Descriptions of phenotypic characteristics of eastern and western Amazonian populations of target species.**
(PDF)
