# Supplementary material for: A Trans-Amazonian Screening of mtDNA Reveals Deep Intraspecific Divergence in Forest Birds and Suggests a Vast Underestimation of Species Diversity
Source: PLoS One. 2012 Jul 17;7(7):e40541. doi: 10.1371/journal.pone.0040541 (PMC3398903; doi:10.1371/journal.pone.0040541)
Supplement: Table S1 — Specimens sampled per species and per locality. Species are listed in alphabetical order. (PDF) [file pone.0040541.s001.pdf]

Table S1. Specimens sampled per species per locality. Species listed in alphabetical order.

ID	Species	Locality	Lat/Long
04N6347	*Automolus ochrolaemus*	Bilsa Biological Station, Bilsa, Esmeraldas, West Ecuador	N 0.36076, W 79.71486
04N6352	*Automolus ochrolaemus*	Bilsa Biological Station, Bilsa, Esmeraldas, West Ecuador	N 0.36076, W 79.71486
05N9842	*Automolus ochrolaemus*	Bilsa Biological Station, Bilsa, Esmeraldas, West Ecuador	N 0.36076, W 79.71486
00N0972	*Automolus ochrolaemus*	Panacocha River, Panacocha, Sucumbios, East Ecuador	S 0.38238, W 76.17612
08-0703	*Automolus ochrolaemus*	Parare Station, Nouragues, French Guiana	N 4.04055, W 52.6757
08-0724	*Automolus ochrolaemus*	Parare Station, Nouragues, French Guiana	N 4.04055, W 52.6757
08-0738	*Automolus ochrolaemus*	Parare Station, Nouragues, French Guiana	N 4.04055, W 52.6757
00N0632	*Campylopterus largipennis*	Chontacocha, Loreto, Orellana, East Ecuador	S 0.67600, W 77.25429
00N0638	*Campylopterus largipennis*	Chontacocha, Loreto, Orellana, East Ecuador	S 0.67600, W 77.25429
00N3305	*Campylopterus largipennis*	Chontacocha, Loreto, Orellana, East Ecuador	S 0.67600, W 77.25429
07-0023	*Campylopterus largipennis*	Inselberg Station, Nouragues, French Guiana	N 4.09412, W 52.68438
07-0091	*Campylopterus largipennis*	Inselberg Station, Nouragues, French Guiana	N 4.09412, W 52.68438
07-0313	*Campylopterus largipennis*	Inselberg Station, Nouragues, French Guiana	N 4.09412, W 52.68438
07-0314	*Campylopterus largipennis*	Inselberg Station, Nouragues, French Guiana	N 4.09412, W 52.68438
07-0355	*Campylopterus largipennis*	Inselberg Station, Nouragues, French Guiana	N 4.09412, W 52.68438
00N3336	*Campylopterus largipennis*	Miazal Mission, Miazal, Morona-Santiago, East Ecuador	S 2.63573, W 77.79831
07-0141	*Chloroceryle aenea*	Inselberg Station, Nouragues, French Guiana	N 4.09412, W 52.68438
00N0667	*Chloroceryle aenea*	Tiputini Biological Station, Tiputini, Orellana, East Ecuador	S 0.63698, W 76.14912
00N1045	*Chloroceryle aenea*	Tiputini Biological Station, Tiputini, Orellana, East Ecuador	S 0.63698, W 76.14912
00N0602	*Conopophaga aurita*	Chontacocha, Loreto, Orellana, East Ecuador	S 0.67600, W 77.25429
00N3254	*Conopophaga aurita*	Chontacocha, Loreto, Orellana, East Ecuador	S 0.67600, W 77.25429
00N3255	*Conopophaga aurita*	Chontacocha, Loreto, Orellana, East Ecuador	S 0.67600, W 77.25429
00N3290	*Conopophaga aurita*	Chontacocha, Loreto, Orellana, East Ecuador	S 0.67600, W 77.25429
00N3291	*Conopophaga aurita*	Chontacocha, Loreto, Orellana, East Ecuador	S 0.67600, W 77.25429
07-0129	*Conopophaga aurita*	Inselberg Station, Nouragues, French Guiana	N 4.09412, W 52.68438
00N0916	*Conopophaga aurita*	Panacocha River, Panacocha, Sucumbios, East Ecuador	S 0.38238, W 76.17612
08-0730	*Conopophaga aurita*	Parare Station, Nouragues, French Guiana	N 4.04055, W 52.6757
07-0101	*Corythopis torquatus*	Inselberg Station, Nouragues, French Guiana	N 4.09412, W 52.68438
07-0103	*Corythopis torquatus*	Inselberg Station, Nouragues, French Guiana	N 4.09412, W 52.68438
07-0128	*Corythopis torquatus*	Inselberg Station, Nouragues, French Guiana	N 4.09412, W 52.68438
07-0130	*Corythopis torquatus*	Inselberg Station, Nouragues, French Guiana	N 4.09412, W 52.68438
99N5247	*Corythopis torquatus*	Jatun Sacha Station, Misahualli, Napo, East Ecuador	S 1.0215, W 77.62144
08-0867	*Corythopis torquatus*	Paracou Field Station, Paracou, French Guiana	N 5.27595, W 52.9280
08-0716	*Corythopis torquatus*	Parare Station, Nouragues, French Guiana	N 4.04055, W 52.6757
08-0729	*Corythopis torquatus*	Parare Station, Nouragues, French Guiana	N 4.04055, W 52.6757
04N5783	*Cyanocompsa cyanoides*	Bilsa Biological Station, Bilsa, Esmeraldas, West Ecuador	N 0.36076, W 79.71486
04N8410	*Cyanocompsa cyanoides*	Bilsa Biological Station, Bilsa, Esmeraldas, West Ecuador	N 0.36076, W 79.71486
05N9237	*Cyanocompsa cyanoides*	Bilsa Biological Station, Bilsa, Esmeraldas, West Ecuador	N 0.36076, W 79.71486
00N0798	*Cyanocompsa cyanoides*	Estacion Cientifica Yasuni, Yasuni, Orellana, East Ecuador	S 0.67455, W 76.39837
07-0037	*Cyanocompsa cyanoides*	Inselberg Station, Nouragues, French Guiana	N 4.09412, W 52.68438
07-0154	*Cyanocompsa cyanoides*	Inselberg Station, Nouragues, French Guiana	N 4.09412, W 52.68438
07-0206	*Cyanocompsa cyanoides*	Inselberg Station, Nouragues, French Guiana	N 4.09412, W 52.68438
99N5259	*Cyanocompsa cyanoides*	Jatun Sacha Station, Misahualli, Napo, East Ecuador	S 1.0215, W 77.62144
99N5260	*Cyanocompsa cyanoides*	Jatun Sacha Station, Misahualli, Napo, East Ecuador	S 1.0215, W 77.62144
00N3332	*Cyanocompsa cyanoides*	Miazal Mission, Miazal, Morona-Santiago, East Ecuador	S 2.63573, W 77.79831
00N3373	*Cyanocompsa cyanoides*	Miazal Mission, Miazal, Morona-Santiago, East Ecuador	S 2.63573, W 77.79831
00N3420	*Cyanocompsa cyanoides*	Miazal Mission, Miazal, Morona-Santiago, East Ecuador	S 2.63573, W 77.79831
00N0689	*Cyanocompsa cyanoides*	Tiputini Biological Station, Tiputini, Orellana, East Ecuador	S 0.63698, W 76.14912
00N5048	*Cyanocompsa cyanoides*	Tiputini Biological Station, Tiputini, Orellana, East Ecuador	S 0.63698, W 76.14912
05N0010	*Cyanocompsa cyanoides*	Tiputini Biological Station, Tiputini, Orellana, East Ecuador	S 0.63698, W 76.14912
00N3310	*Cyphorhinus arada*	Chontacocha, Loreto, Orellana, East Ecuador	S 0.67600, W 77.25429
00N0517	*Cyphorhinus arada*	Hollin River, Hollin, Napo, East Ecuador	S 0.68896, W 77.72658
07-0102	*Cyphorhinus arada*	Inselberg Station, Nouragues, French Guiana	N 4.09412, W 52.68438
07-0104	*Cyphorhinus arada*	Inselberg Station, Nouragues, French Guiana	N 4.09412, W 52.68438
07-0109	*Cyphorhinus arada*	Inselberg Station, Nouragues, French Guiana	N 4.09412, W 52.68438
07-0110	*Cyphorhinus arada*	Inselberg Station, Nouragues, French Guiana	N 4.09412, W 52.68438
08-0761	*Cyphorhinus arada*	Parare Station, Nouragues, French Guiana	N 4.04055, W 52.6757
04N5692	*Dendrocincla fuliginosa*	Bilsa Biological Station, Bilsa, Esmeraldas, West Ecuador	N 0.36076, W 79.71486
04N5698	*Dendrocincla fuliginosa*	Bilsa Biological Station, Bilsa, Esmeraldas, West Ecuador	N 0.36076, W 79.71486
04N5727	*Dendrocincla fuliginosa*	Bilsa Biological Station, Bilsa, Esmeraldas, West Ecuador	N 0.36076, W 79.71486
04N5728	*Dendrocincla fuliginosa*	Bilsa Biological Station, Bilsa, Esmeraldas, West Ecuador	N 0.36076, W 79.71486
04N5837	*Dendrocincla fuliginosa*	Bilsa Biological Station, Bilsa, Esmeraldas, West Ecuador	N 0.36076, W 79.71486
04N5873	*Dendrocincla fuliginosa*	Bilsa Biological Station, Bilsa, Esmeraldas, West Ecuador	N 0.36076, W 79.71486
04N5907	*Dendrocincla fuliginosa*	Bilsa Biological Station, Bilsa, Esmeraldas, West Ecuador	N 0.36076, W 79.71486
04N5913	*Dendrocincla fuliginosa*	Bilsa Biological Station, Bilsa, Esmeraldas, West Ecuador	N 0.36076, W 79.71486
04N5915	*Dendrocincla fuliginosa*	Bilsa Biological Station, Bilsa, Esmeraldas, West Ecuador	N 0.36076, W 79.71486
04N5962	*Dendrocincla fuliginosa*	Bilsa Biological Station, Bilsa, Esmeraldas, West Ecuador	N 0.36076, W 79.71486
04N5988	*Dendrocincla fuliginosa*	Bilsa Biological Station, Bilsa, Esmeraldas, West Ecuador	N 0.36076, W 79.71486
04N6024	*Dendrocincla fuliginosa*	Bilsa Biological Station, Bilsa, Esmeraldas, West Ecuador	N 0.36076, W 79.71486
06N1706	*Dendrocincla fuliginosa*	Bilsa Biological Station, Bilsa, Esmeraldas, West Ecuador	N 0.36076, W 79.71486
00N0621	*Dendrocincla fuliginosa*	Chontacocha, Loreto, Orellana, East Ecuador	S 0.67600, W 77.25429
00N3275	*Dendrocincla fuliginosa*	Chontacocha, Loreto, Orellana, East Ecuador	S 0.67600, W 77.25429
00N3230	*Dendrocincla fuliginosa*	Hollin River, Hollin, Napo, East Ecuador	S 0.68896, W 77.72658
07-0215	*Dendrocincla fuliginosa*	Inselberg Station, Nouragues, French Guiana	N 4.09412, W 52.68438
07-0227	*Dendrocincla fuliginosa*	Inselberg Station, Nouragues, French Guiana	N 4.09412, W 52.68438
00N0995	*Dendrocincla fuliginosa*	Panacocha River, Panacocha, Sucumbios, East Ecuador	S 0.38238, W 76.17612
00N1007	*Dendrocincla fuliginosa*	Panacocha River, Panacocha, Sucumbios, East Ecuador	S 0.38238, W 76.17612
00N1034	*Dendrocincla fuliginosa*	Panacocha River, Panacocha, Sucumbios, East Ecuador	S 0.38238, W 76.17612
08-0823	*Dendrocincla fuliginosa*	Paracou Field Station, Paracou, French Guiana	N 5.27595, W 52.9280
08-0824	*Dendrocincla fuliginosa*	Paracou Field Station, Paracou, French Guiana	N 5.27595, W 52.9280
00N5067	*Dendrocincla fuliginosa*	Tiputini Biological Station, Tiputini, Orellana, East Ecuador	S 0.63698, W 76.14912
00N0885	*Dixiphia pipra*	Estacion Cientifica Yasuni, Yasuni, Orellana, East Ecuador	S 0.67455, W 76.39837
00N0570	*Dixiphia pipra*	Guagua Sumaco, Guagua Sumaco, Napo, East Ecuador	S 0.72493, W 77.57564
00N0577	*Dixiphia pipra*	Guagua Sumaco, Guagua Sumaco, Napo, East Ecuador	S 0.72493, W 77.57564
00N0591	*Dixiphia pipra*	Guagua Sumaco, Guagua Sumaco, Napo, East Ecuador	S 0.72493, W 77.57564
00N3175	*Dixiphia pipra*	Hollin River, Hollin, Napo, East Ecuador	S 0.68896, W 77.72658
00N3218	*Dixiphia pipra*	Hollin River, Hollin, Napo, East Ecuador	S 0.68896, W 77.72658
00N3226	*Dixiphia pipra*	Hollin River, Hollin, Napo, East Ecuador	S 0.68896, W 77.72658

00N3232	*Dixiphia pipra*	Hollin River, Hollin, Napo, East Ecuador	S 0.68896, W 77.72658
00N3235	*Dixiphia pipra*	Hollin River, Hollin, Napo, East Ecuador	S 0.68896, W 77.72658
07-0016	*Dixiphia pipra*	Inselberg Station, Nouragues, French Guiana	N 4.09412, W 52.68438
07-0026	*Dixiphia pipra*	Inselberg Station, Nouragues, French Guiana	N 4.09412, W 52.68438
07-0044	*Dixiphia pipra*	Inselberg Station, Nouragues, French Guiana	N 4.09412, W 52.68438
07-0049	*Dixiphia pipra*	Inselberg Station, Nouragues, French Guiana	N 4.09412, W 52.68438
07-0054	*Dixiphia pipra*	Inselberg Station, Nouragues, French Guiana	N 4.09412, W 52.68438
07-0162	*Dixiphia pipra*	Inselberg Station, Nouragues, French Guiana	N 4.09412, W 52.68438
07-0163	*Dixiphia pipra*	Inselberg Station, Nouragues, French Guiana	N 4.09412, W 52.68438
07-0172	*Dixiphia pipra*	Inselberg Station, Nouragues, French Guiana	N 4.09412, W 52.68438
07-0212	*Dixiphia pipra*	Inselberg Station, Nouragues, French Guiana	N 4.09412, W 52.68438
07-0299	*Dixiphia pipra*	Inselberg Station, Nouragues, French Guiana	N 4.09412, W 52.68438
07-0302	*Dixiphia pipra*	Inselberg Station, Nouragues, French Guiana	N 4.09412, W 52.68438
07-0303	*Dixiphia pipra*	Inselberg Station, Nouragues, French Guiana	N 4.09412, W 52.68438
07-0323	*Dixiphia pipra*	Inselberg Station, Nouragues, French Guiana	N 4.09412, W 52.68438
00N3474	*Dixiphia pipra*	Nueva Alianza, Sangay National Park, Morona-Santiago, East Ecuador	S 2.09883, W 78.15164
00N3475	*Dixiphia pipra*	Nueva Alianza, Sangay National Park, Morona-Santiago, East Ecuador	S 2.09883, W 78.15164
00N3478	*Dixiphia pipra*	Nueva Alianza, Sangay National Park, Morona-Santiago, East Ecuador	S 2.09883, W 78.15164
00N3498	*Dixiphia pipra*	Nueva Alianza, Sangay National Park, Morona-Santiago, East Ecuador	S 2.09883, W 78.15164
00N3502	*Dixiphia pipra*	Nueva Alianza, Sangay National Park, Morona-Santiago, East Ecuador	S 2.09883, W 78.15164
00N3521	*Dixiphia pipra*	Nueva Alianza, Sangay National Park, Morona-Santiago, East Ecuador	S 2.09883, W 78.15164
00N3539	*Dixiphia pipra*	Nueva Alianza, Sangay National Park, Morona-Santiago, East Ecuador	S 2.09883, W 78.15164
08-0834	*Dixiphia pipra*	Paracou Field Station, Paracou, French Guiana	N 5.27595, W 52.9280
08-0835	*Dixiphia pipra*	Paracou Field Station, Paracou, French Guiana	N 5.27595, W 52.9280
08-0836	*Dixiphia pipra*	Paracou Field Station, Paracou, French Guiana	N 5.27595, W 52.9280
08-0843	*Dixiphia pipra*	Paracou Field Station, Paracou, French Guiana	N 5.27595, W 52.9280
08-0853	*Dixiphia pipra*	Paracou Field Station, Paracou, French Guiana	N 5.27595, W 52.9280
08-0864	*Dixiphia pipra*	Paracou Field Station, Paracou, French Guiana	N 5.27595, W 52.9280
08-0884	*Dixiphia pipra*	Paracou Field Station, Paracou, French Guiana	N 5.27595, W 52.9280
08-0898	*Dixiphia pipra*	Paracou Field Station, Paracou, French Guiana	N 5.27595, W 52.9280
08-0899	*Dixiphia pipra*	Paracou Field Station, Paracou, French Guiana	N 5.27595, W 52.9280
08-0603	*Dixiphia pipra*	Parare Station, Nouragues, French Guiana	N 4.04055, W 52.6757
08-0622	*Dixiphia pipra*	Parare Station, Nouragues, French Guiana	N 4.04055, W 52.6757
08-0647	*Dixiphia pipra*	Parare Station, Nouragues, French Guiana	N 4.04055, W 52.6757
08-0736	*Dixiphia pipra*	Parare Station, Nouragues, French Guiana	N 4.04055, W 52.6757
00N0785	*Dixiphia pipra*	Tiputini Biological Station, Tiputini, Orellana, East Ecuador	S 0.63698, W 76.14912
00N5025	*Dixiphia pipra*	Tiputini Biological Station, Tiputini, Orellana, East Ecuador	S 0.63698, W 76.14912
05N0058	*Dixiphia pipra*	Tiputini Biological Station, Tiputini, Orellana, East Ecuador	S 0.63698, W 76.14912
00N0605	*Formicarius analis*	Chontacocha, Loreto, Orellana, East Ecuador	S 0.67600, W 77.25429
00N3261	*Formicarius analis*	Chontacocha, Loreto, Orellana, East Ecuador	S 0.67600, W 77.25429
00N3292	*Formicarius analis*	Chontacocha, Loreto, Orellana, East Ecuador	S 0.67600, W 77.25429
07-0132	*Formicarius analis*	Inselberg Station, Nouragues, French Guiana	N 4.09412, W 52.68438
00N3444	*Formicarius analis*	Miazal Mission, Miazal, Morona-Santiago, East Ecuador	S 2.63573, W 77.79831
05N0012	*Formicarius analis*	Tiputini Biological Station, Tiputini, Orellana, East Ecuador	S 0.63698, W 76.14912
00N0809	*Formicarius colma*	Estacion Cientifica Yasuni, Yasuni, Orellana, East Ecuador	S 0.67455, W 76.39837
00N0849	*Formicarius colma*	Estacion Cientifica Yasuni, Yasuni, Orellana, East Ecuador	S 0.67455, W 76.39837
07-0105	*Formicarius colma*	Inselberg Station, Nouragues, French Guiana	N 4.09412, W 52.68438
07-0111	*Formicarius colma*	Inselberg Station, Nouragues, French Guiana	N 4.09412, W 52.68438
07-0237	*Formicarius colma*	Inselberg Station, Nouragues, French Guiana	N 4.09412, W 52.68438
07-0240	*Formicarius colma*	Inselberg Station, Nouragues, French Guiana	N 4.09412, W 52.68438
07-0359	*Formicarius colma*	Inselberg Station, Nouragues, French Guiana	N 4.09412, W 52.68438
07-0360	*Formicarius colma*	Inselberg Station, Nouragues, French Guiana	N 4.09412, W 52.68438
00N0907	*Formicarius colma*	Panacocha River, Panacocha, Sucumbios, East Ecuador	S 0.38238, W 76.17612
00N0938	*Formicarius colma*	Panacocha River, Panacocha, Sucumbios, East Ecuador	S 0.38238, W 76.17612
00N0974	*Formicarius colma*	Panacocha River, Panacocha, Sucumbios, East Ecuador	S 0.38238, W 76.17612
08-0666	*Formicarius colma*	Parare Station, Nouragues, French Guiana	N 4.04055, W 52.6757
08-0735	*Formicarius colma*	Parare Station, Nouragues, French Guiana	N 4.04055, W 52.6757
08-0801	*Formicarius colma*	Parare Station, Nouragues, French Guiana	N 4.04055, W 52.6757
00N0759	*Formicarius colma*	Tiputini Biological Station, Tiputini, Orellana, East Ecuador	S 0.63698, W 76.14912
07-0019	*Galbula albirostris*	Inselberg Station, Nouragues, French Guiana	N 4.09412, W 52.68438
07-0021	*Galbula albirostris*	Inselberg Station, Nouragues, French Guiana	N 4.09412, W 52.68438
07-0027	*Galbula albirostris*	Inselberg Station, Nouragues, French Guiana	N 4.09412, W 52.68438
07-0092	*Galbula albirostris*	Inselberg Station, Nouragues, French Guiana	N 4.09412, W 52.68438
07-0094	*Galbula albirostris*	Inselberg Station, Nouragues, French Guiana	N 4.09412, W 52.68438
00N5010	*Galbula albirostris*	Tiputini Biological Station, Tiputini, Orellana, East Ecuador	S 0.63698, W 76.14912
00N5037	*Galbula albirostris*	Tiputini Biological Station, Tiputini, Orellana, East Ecuador	S 0.63698, W 76.14912
05N0042	*Galbula albirostris*	Tiputini Biological Station, Tiputini, Orellana, East Ecuador	S 0.63698, W 76.14912
04N8318	*Geotrygon montana*	Bilsa Biological Station, Bilsa, Esmeraldas, West Ecuador	N 0.36076, W 79.71486
00N0619	*Geotrygon montana*	Chontacocha, Loreto, Orellana, East Ecuador	S 0.67600, W 77.25429
07-0048	*Geotrygon montana*	Inselberg Station, Nouragues, French Guiana	N 4.09412, W 52.68438
07-0306	*Geotrygon montana*	Inselberg Station, Nouragues, French Guiana	N 4.09412, W 52.68438
99N5257	*Geotrygon montana*	Jatun Sacha Station, Misahualli, Napo, East Ecuador	S 1.0215, W 77.62144
00N3493	*Geotrygon montana*	Nueva Alianza, Sangay National Park, Morona-Santiago, East Ecuador	S 2.09883, W 78.15164
00N3528	*Geotrygon montana*	Nueva Alianza, Sangay National Park, Morona-Santiago, East Ecuador	S 2.09883, W 78.15164
05N0029	*Geotrygon montana*	Tiputini Biological Station, Tiputini, Orellana, East Ecuador	S 0.63698, W 76.14912
03N1726	*Glyphorynchus spirurus*	Bilsa Biological Station, Bilsa, Esmeraldas, West Ecuador	N 0.36076, W 79.71486
03N1757	*Glyphorynchus spirurus*	Bilsa Biological Station, Bilsa, Esmeraldas, West Ecuador	N 0.36076, W 79.71486
03N1780	*Glyphorynchus spirurus*	Bilsa Biological Station, Bilsa, Esmeraldas, West Ecuador	N 0.36076, W 79.71486
03N1782	*Glyphorynchus spirurus*	Bilsa Biological Station, Bilsa, Esmeraldas, West Ecuador	N 0.36076, W 79.71486
03N1783	*Glyphorynchus spirurus*	Bilsa Biological Station, Bilsa, Esmeraldas, West Ecuador	N 0.36076, W 79.71486
03N1784	*Glyphorynchus spirurus*	Bilsa Biological Station, Bilsa, Esmeraldas, West Ecuador	N 0.36076, W 79.71486
03N1785	*Glyphorynchus spirurus*	Bilsa Biological Station, Bilsa, Esmeraldas, West Ecuador	N 0.36076, W 79.71486
03N1817	*Glyphorynchus spirurus*	Bilsa Biological Station, Bilsa, Esmeraldas, West Ecuador	N 0.36076, W 79.71486
03N1825	*Glyphorynchus spirurus*	Bilsa Biological Station, Bilsa, Esmeraldas, West Ecuador	N 0.36076, W 79.71486
03N1850	*Glyphorynchus spirurus*	Bilsa Biological Station, Bilsa, Esmeraldas, West Ecuador	N 0.36076, W 79.71486
02N9159	*Glyphorynchus spirurus*	Cumanda, Cumanda, Pastaza, East Ecuador	S 1.47686, W 78.14325
02N9171	*Glyphorynchus spirurus*	Cumanda, Cumanda, Pastaza, East Ecuador	S 1.47686, W 78.14325
02N9176	*Glyphorynchus spirurus*	Cumanda, Cumanda, Pastaza, East Ecuador	S 1.47686, W 78.14325
02N9178	*Glyphorynchus spirurus*	Cumanda, Cumanda, Pastaza, East Ecuador	S 1.47686, W 78.14325
02N9184	*Glyphorynchus spirurus*	Cumanda, Cumanda, Pastaza, East Ecuador	S 1.47686, W 78.14325
07-0011	*Glyphorynchus spirurus*	Inselberg Station, Nouragues, French Guiana	N 4.09412, W 52.68438
07-0017	*Glyphorynchus spirurus*	Inselberg Station, Nouragues, French Guiana	N 4.09412, W 52.68438

07-0025	*Glyphorynchus spirurus*	Inselberg Station, Nouragues, French Guiana	N 4.09412, W 52.68438
07-0035	*Glyphorynchus spirurus*	Inselberg Station, Nouragues, French Guiana	N 4.09412, W 52.68438
07-0045	*Glyphorynchus spirurus*	Inselberg Station, Nouragues, French Guiana	N 4.09412, W 52.68438
07-0055	*Glyphorynchus spirurus*	Inselberg Station, Nouragues, French Guiana	N 4.09412, W 52.68438
07-0203	*Glyphorynchus spirurus*	Inselberg Station, Nouragues, French Guiana	N 4.09412, W 52.68438
07-0205	*Glyphorynchus spirurus*	Inselberg Station, Nouragues, French Guiana	N 4.09412, W 52.68438
07-0224	*Glyphorynchus spirurus*	Inselberg Station, Nouragues, French Guiana	N 4.09412, W 52.68438
07-0235	*Glyphorynchus spirurus*	Inselberg Station, Nouragues, French Guiana	N 4.09412, W 52.68438
07-0346	*Glyphorynchus spirurus*	Inselberg Station, Nouragues, French Guiana	N 4.09412, W 52.68438
07-0361	*Glyphorynchus spirurus*	Inselberg Station, Nouragues, French Guiana	N 4.09412, W 52.68438
03N1563	*Glyphorynchus spirurus*	Loma Alta, Chongon-Colonche, Guayas, West Ecuador	S 1.83475, W 80.61143
03N1570	*Glyphorynchus spirurus*	Loma Alta, Chongon-Colonche, Guayas, West Ecuador	S 1.83475, W 80.61143
03N1603	*Glyphorynchus spirurus*	Loma Alta, Chongon-Colonche, Guayas, West Ecuador	S 1.83475, W 80.61143
03N1652	*Glyphorynchus spirurus*	Loma Alta, Chongon-Colonche, Guayas, West Ecuador	S 1.83475, W 80.61143
03N1654	*Glyphorynchus spirurus*	Loma Alta, Chongon-Colonche, Guayas, West Ecuador	S 1.83475, W 80.61143
02N9256	*Glyphorynchus spirurus*	Nueva Alianza, Sangay National Park, Morona-Santiago, East Ecuador	S 2.09883, W 78.15164
02N9257	*Glyphorynchus spirurus*	Nueva Alianza, Sangay National Park, Morona-Santiago, East Ecuador	S 2.09883, W 78.15164
02N9258	*Glyphorynchus spirurus*	Nueva Alianza, Sangay National Park, Morona-Santiago, East Ecuador	S 2.09883, W 78.15164
02N9264	*Glyphorynchus spirurus*	Nueva Alianza, Sangay National Park, Morona-Santiago, East Ecuador	S 2.09883, W 78.15164
02N9301	*Glyphorynchus spirurus*	Nueva Alianza, Sangay National Park, Morona-Santiago, East Ecuador	S 2.09883, W 78.15164
00N0922	*Glyphorynchus spirurus*	Panacocha River, Panacocha, Sucumbios, East Ecuador	S 0.38238, W 76.17612
00N0923	*Glyphorynchus spirurus*	Panacocha River, Panacocha, Sucumbios, East Ecuador	S 0.38238, W 76.17612
00N0965	*Glyphorynchus spirurus*	Panacocha River, Panacocha, Sucumbios, East Ecuador	S 0.38238, W 76.17612
00N0980	*Glyphorynchus spirurus*	Panacocha River, Panacocha, Sucumbios, East Ecuador	S 0.38238, W 76.17612
00N0981	*Glyphorynchus spirurus*	Panacocha River, Panacocha, Sucumbios, East Ecuador	S 0.38238, W 76.17612
08-0837	*Glyphorynchus spirurus*	Paracou Field Station, Paracou, French Guiana	N 5.27595, W 52.9280
08-0870	*Glyphorynchus spirurus*	Paracou Field Station, Paracou, French Guiana	N 5.27595, W 52.9280
08-0875	*Glyphorynchus spirurus*	Paracou Field Station, Paracou, French Guiana	N 5.27595, W 52.9280
08-0888	*Glyphorynchus spirurus*	Paracou Field Station, Paracou, French Guiana	N 5.27595, W 52.9280
08-0902	*Glyphorynchus spirurus*	Paracou Field Station, Paracou, French Guiana	N 5.27595, W 52.9280
08-0768	*Glyphorynchus spirurus*	Parare Station, Nouragues, French Guiana	N 4.04055, W 52.6757
08-0787	*Glyphorynchus spirurus*	Parare Station, Nouragues, French Guiana	N 4.04055, W 52.6757
08-0794	*Glyphorynchus spirurus*	Parare Station, Nouragues, French Guiana	N 4.04055, W 52.6757
08-0796	*Glyphorynchus spirurus*	Parare Station, Nouragues, French Guiana	N 4.04055, W 52.6757
08-0812	*Glyphorynchus spirurus*	Parare Station, Nouragues, French Guiana	N 4.04055, W 52.6757
00N0669	*Glyphorynchus spirurus*	Tiputini Biological Station, Tiputini, Orellana, East Ecuador	S 0.63698, W 76.14912
00N0670	*Glyphorynchus spirurus*	Tiputini Biological Station, Tiputini, Orellana, East Ecuador	S 0.63698, W 76.14912
00N0671	*Glyphorynchus spirurus*	Tiputini Biological Station, Tiputini, Orellana, East Ecuador	S 0.63698, W 76.14912
00N0685	*Glyphorynchus spirurus*	Tiputini Biological Station, Tiputini, Orellana, East Ecuador	S 0.63698, W 76.14912
00N0704	*Glyphorynchus spirurus*	Tiputini Biological Station, Tiputini, Orellana, East Ecuador	S 0.63698, W 76.14912
00N3269	*Hylophilus ochraceiceps*	Chontacocha, Loreto, Orellana, East Ecuador	S 0.67600, W 77.25429
00N0863	*Hylophilus ochraceiceps*	Estacion Cientifica Yasuni, Yasuni, Orellana, East Ecuador	S 0.67455, W 76.39837
07-0084	*Hylophilus ochraceiceps*	Inselberg Station, Nouragues, French Guiana	N 4.09412, W 52.68438
08-0877	*Hylophilus ochraceiceps*	Paracou Field Station, Paracou, French Guiana	N 5.27595, W 52.9280
00N0724	*Hylophilus ochraceiceps*	Tiputini Biological Station, Tiputini, Orellana, East Ecuador	S 0.63698, W 76.14912
00N0731	*Hylophilus ochraceiceps*	Tiputini Biological Station, Tiputini, Orellana, East Ecuador	S 0.63698, W 76.14912
05N0030	*Hylophilus ochraceiceps*	Tiputini Biological Station, Tiputini, Orellana, East Ecuador	S 0.63698, W 76.14912
05N0035	*Hylophilus ochraceiceps*	Tiputini Biological Station, Tiputini, Orellana, East Ecuador	S 0.63698, W 76.14912
02N9157	*Hylophylax naevius*	Cumanda, Cumanda, Pastaza, East Ecuador	S 1.47686, W 78.14325
02N9180	*Hylophylax naevius*	Cumanda, Cumanda, Pastaza, East Ecuador	S 1.47686, W 78.14325
00N0552	*Hylophylax naevius*	Guagua Sumaco, Guagua Sumaco, Napo, East Ecuador	S 0.72493, W 77.57564
00N0553	*Hylophylax naevius*	Guagua Sumaco, Guagua Sumaco, Napo, East Ecuador	S 0.72493, W 77.57564
00N0583	*Hylophylax naevius*	Guagua Sumaco, Guagua Sumaco, Napo, East Ecuador	S 0.72493, W 77.57564
00N0584	*Hylophylax naevius*	Guagua Sumaco, Guagua Sumaco, Napo, East Ecuador	S 0.72493, W 77.57564
00N0503	*Hylophylax naevius*	Hollin River, Hollin, Napo, East Ecuador	S 0.68896, W 77.72658
00N0509	*Hylophylax naevius*	Hollin River, Hollin, Napo, East Ecuador	S 0.68896, W 77.72658
00N0530	*Hylophylax naevius*	Hollin River, Hollin, Napo, East Ecuador	S 0.68896, W 77.72658
00N3181	*Hylophylax naevius*	Hollin River, Hollin, Napo, East Ecuador	S 0.68896, W 77.72658
07-0003	*Hylophylax naevius*	Inselberg Station, Nouragues, French Guiana	N 4.09412, W 52.68438
07-0036	*Hylophylax naevius*	Inselberg Station, Nouragues, French Guiana	N 4.09412, W 52.68438
07-0140	*Hylophylax naevius*	Inselberg Station, Nouragues, French Guiana	N 4.09412, W 52.68438
07-0208	*Hylophylax naevius*	Inselberg Station, Nouragues, French Guiana	N 4.09412, W 52.68438
07-0255	*Hylophylax naevius*	Inselberg Station, Nouragues, French Guiana	N 4.09412, W 52.68438
07-0257	*Hylophylax naevius*	Inselberg Station, Nouragues, French Guiana	N 4.09412, W 52.68438
07-0296	*Hylophylax naevius*	Inselberg Station, Nouragues, French Guiana	N 4.09412, W 52.68438
00N3398	*Hylophylax naevius*	Miazal Mission, Miazal, Morona-Santiago, East Ecuador	S 2.63573, W 77.79831
00N3409	*Hylophylax naevius*	Miazal Mission, Miazal, Morona-Santiago, East Ecuador	S 2.63573, W 77.79831
08-0613	*Hylophylax naevius*	Parare Station, Nouragues, French Guiana	N 4.04055, W 52.6757
08-0615	*Hylophylax naevius*	Parare Station, Nouragues, French Guiana	N 4.04055, W 52.6757
08-0670	*Hylophylax naevius*	Parare Station, Nouragues, French Guiana	N 4.04055, W 52.6757
08-0748	*Hylophylax naevius*	Parare Station, Nouragues, French Guiana	N 4.04055, W 52.6757
00N0728	*Hylophylax naevius*	Tiputini Biological Station, Tiputini, Orellana, East Ecuador	S 0.63698, W 76.14912
00N5013	*Hylophylax naevius*	Tiputini Biological Station, Tiputini, Orellana, East Ecuador	S 0.63698, W 76.14912
00N5017	*Hylophylax naevius*	Tiputini Biological Station, Tiputini, Orellana, East Ecuador	S 0.63698, W 76.14912
00N5045	*Hylophylax naevius*	Tiputini Biological Station, Tiputini, Orellana, East Ecuador	S 0.63698, W 76.14912
00N5055	*Hylophylax naevius*	Tiputini Biological Station, Tiputini, Orellana, East Ecuador	S 0.63698, W 76.14912
00N5063	*Hylophylax naevius*	Tiputini Biological Station, Tiputini, Orellana, East Ecuador	S 0.63698, W 76.14912
05N0045	*Hylophylax naevius*	Tiputini Biological Station, Tiputini, Orellana, East Ecuador	S 0.63698, W 76.14912
05N0091	*Hylophylax naevius*	Tiputini Biological Station, Tiputini, Orellana, East Ecuador	S 0.63698, W 76.14912
00N0799	*Hylophylax poecilinotus*	Estacion Cientifica Yasuni, Yasuni, Orellana, East Ecuador	S 0.67455, W 76.39837
00N0839	*Hylophylax poecilinotus*	Estacion Cientifica Yasuni, Yasuni, Orellana, East Ecuador	S 0.67455, W 76.39837
00N0895	*Hylophylax poecilinotus*	Estacion Cientifica Yasuni, Yasuni, Orellana, East Ecuador	S 0.67455, W 76.39837
00N0550	*Hylophylax poecilinotus*	Guagua Sumaco, Guagua Sumaco, Napo, East Ecuador	S 0.72493, W 77.57564
00N0594	*Hylophylax poecilinotus*	Guagua Sumaco, Guagua Sumaco, Napo, East Ecuador	S 0.72493, W 77.57564
00N3219	*Hylophylax poecilinotus*	Hollin River, Hollin, Napo, East Ecuador	S 0.68896, W 77.72658
00N3222	*Hylophylax poecilinotus*	Hollin River, Hollin, Napo, East Ecuador	S 0.68896, W 77.72658
02N9081	*Hylophylax poecilinotus*	Hollin River, Hollin, Napo, East Ecuador	S 0.68896, W 77.72658
07-0031	*Hylophylax poecilinotus*	Inselberg Station, Nouragues, French Guiana	N 4.09412, W 52.68438
07-0032	*Hylophylax poecilinotus*	Inselberg Station, Nouragues, French Guiana	N 4.09412, W 52.68438
07-0033	*Hylophylax poecilinotus*	Inselberg Station, Nouragues, French Guiana	N 4.09412, W 52.68438
07-0056	*Hylophylax poecilinotus*	Inselberg Station, Nouragues, French Guiana	N 4.09412, W 52.68438
07-0063	*Hylophylax poecilinotus*	Inselberg Station, Nouragues, French Guiana	N 4.09412, W 52.68438

07-0089	*Hylophylax poecilinotus*	Inselberg Station, Nouragues, French Guiana	N 4.09412, W 52.68438
07-0243	*Hylophylax poecilinotus*	Inselberg Station, Nouragues, French Guiana	N 4.09412, W 52.68438
07-0261	*Hylophylax poecilinotus*	Inselberg Station, Nouragues, French Guiana	N 4.09412, W 52.68438
07-0297	*Hylophylax poecilinotus*	Inselberg Station, Nouragues, French Guiana	N 4.09412, W 52.68438
07-0318	*Hylophylax poecilinotus*	Inselberg Station, Nouragues, French Guiana	N 4.09412, W 52.68438
07-0321	*Hylophylax poecilinotus*	Inselberg Station, Nouragues, French Guiana	N 4.09412, W 52.68438
07-0329	*Hylophylax poecilinotus*	Inselberg Station, Nouragues, French Guiana	N 4.09412, W 52.68438
00N3331	*Hylophylax poecilinotus*	Miazal Mission, Miazal, Morona-Santiago, East Ecuador	S 2.63573, W 77.79831
00N3343	*Hylophylax poecilinotus*	Miazal Mission, Miazal, Morona-Santiago, East Ecuador	S 2.63573, W 77.79831
00N0909	*Hylophylax poecilinotus*	Panacocha River, Panacocha, Sucumbios, East Ecuador	S 0.38238, W 76.17612
00N0942	*Hylophylax poecilinotus*	Panacocha River, Panacocha, Sucumbios, East Ecuador	S 0.38238, W 76.17612
08-0832	*Hylophylax poecilinotus*	Paracou Field Station, Paracou, French Guiana	N 5.27595, W 52.9280
08-0833	*Hylophylax poecilinotus*	Paracou Field Station, Paracou, French Guiana	N 5.27595, W 52.9280
08-0839	*Hylophylax poecilinotus*	Paracou Field Station, Paracou, French Guiana	N 5.27595, W 52.9280
08-0891	*Hylophylax poecilinotus*	Paracou Field Station, Paracou, French Guiana	N 5.27595, W 52.9280
08-0663	*Hylophylax poecilinotus*	Parare Station, Nouragues, French Guiana	N 4.04055, W 52.6757
08-0664	*Hylophylax poecilinotus*	Parare Station, Nouragues, French Guiana	N 4.04055, W 52.6757
08-0779	*Hylophylax poecilinotus*	Parare Station, Nouragues, French Guiana	N 4.04055, W 52.6757
00N0677	*Hylophylax poecilinotus*	Tiputini Biological Station, Tiputini, Orellana, East Ecuador	S 0.63698, W 76.14912
00N0691	*Hylophylax poecilinotus*	Tiputini Biological Station, Tiputini, Orellana, East Ecuador	S 0.63698, W 76.14912
00N0699	*Hylophylax poecilinotus*	Tiputini Biological Station, Tiputini, Orellana, East Ecuador	S 0.63698, W 76.14912
00N0763	*Hylophylax poecilinotus*	Tiputini Biological Station, Tiputini, Orellana, East Ecuador	S 0.63698, W 76.14912
00N0765	*Hylophylax poecilinotus*	Tiputini Biological Station, Tiputini, Orellana, East Ecuador	S 0.63698, W 76.14912
00N0768	*Hylophylax poecilinotus*	Tiputini Biological Station, Tiputini, Orellana, East Ecuador	S 0.63698, W 76.14912
00N0769	*Hylophylax poecilinotus*	Tiputini Biological Station, Tiputini, Orellana, East Ecuador	S 0.63698, W 76.14912
00N5001	*Hylophylax poecilinotus*	Tiputini Biological Station, Tiputini, Orellana, East Ecuador	S 0.63698, W 76.14912
00N5020	*Hylophylax poecilinotus*	Tiputini Biological Station, Tiputini, Orellana, East Ecuador	S 0.63698, W 76.14912
00N5021	*Hylophylax poecilinotus*	Tiputini Biological Station, Tiputini, Orellana, East Ecuador	S 0.63698, W 76.14912
00N5047	*Hylophylax poecilinotus*	Tiputini Biological Station, Tiputini, Orellana, East Ecuador	S 0.63698, W 76.14912
05N0003	*Hylophylax poecilinotus*	Tiputini Biological Station, Tiputini, Orellana, East Ecuador	S 0.63698, W 76.14912
05N0008	*Hylophylax poecilinotus*	Tiputini Biological Station, Tiputini, Orellana, East Ecuador	S 0.63698, W 76.14912
05N0074	*Hylophylax poecilinotus*	Tiputini Biological Station, Tiputini, Orellana, East Ecuador	S 0.63698, W 76.14912
00N3250	*Hypocnemis cantator*	Chontacocha, Loreto, Orellana, East Ecuador	S 0.67600, W 77.25429
07-0139	*Hypocnemis cantator*	Inselberg Station, Nouragues, French Guiana	N 4.09412, W 52.68438
07-0179	*Hypocnemis cantator*	Inselberg Station, Nouragues, French Guiana	N 4.09412, W 52.68438
07-0234	*Hypocnemis cantator*	Inselberg Station, Nouragues, French Guiana	N 4.09412, W 52.68438
07-0276	*Hypocnemis cantator*	Inselberg Station, Nouragues, French Guiana	N 4.09412, W 52.68438
99N5228	*Hypocnemis cantator*	Jatun Sacha Station, Misahualli, Napo, East Ecuador	S 1.0215, W 77.62144
00N3454	*Hypocnemis cantator*	Miazal Mission, Miazal, Morona-Santiago, East Ecuador	S 2.63573, W 77.79831
08-0608	*Hypocnemis cantator*	Parare Station, Nouragues, French Guiana	N 4.04055, W 52.6757
08-0788	*Hypocnemis cantator*	Parare Station, Nouragues, French Guiana	N 4.04055, W 52.6757
00N0611	*Lanio fulvus*	Chontacocha, Loreto, Orellana, East Ecuador	S 0.67600, W 77.25429
07-0099	*Lanio fulvus*	Inselberg Station, Nouragues, French Guiana	N 4.09412, W 52.68438
08-0880	*Lanio fulvus*	Paracou Field Station, Paracou, French Guiana	N 5.27595, W 52.9280
08-0882	*Lanio fulvus*	Paracou Field Station, Paracou, French Guiana	N 5.27595, W 52.9280
00N0705	*Lanio fulvus*	Tiputini Biological Station, Tiputini, Orellana, East Ecuador	S 0.63698, W 76.14912
00N3258	*Microcerculus bambla*	Chontacocha, Loreto, Orellana, East Ecuador	S 0.67600, W 77.25429
00N0586	*Microcerculus bambla*	Guagua Sumaco, Guagua Sumaco, Napo, East Ecuador	S 0.72493, W 77.57564
07-0113	*Microcerculus bambla*	Inselberg Station, Nouragues, French Guiana	N 4.09412, W 52.68438
07-0171	*Microcerculus bambla*	Inselberg Station, Nouragues, French Guiana	N 4.09412, W 52.68438
02N9314	*Microcerculus bambla*	Nueva Alianza, Sangay National Park, Morona-Santiago, East Ecuador	S 2.09883, W 78.15164
07-0005	*Mionectes macconelli*	Inselberg Station, Nouragues, French Guiana	N 4.09412, W 52.68438
07-0014	*Mionectes macconelli*	Inselberg Station, Nouragues, French Guiana	N 4.09412, W 52.68438
07-0061	*Mionectes macconelli*	Inselberg Station, Nouragues, French Guiana	N 4.09412, W 52.68438
07-0069	*Mionectes macconelli*	Inselberg Station, Nouragues, French Guiana	N 4.09412, W 52.68438
07-0072	*Mionectes macconelli*	Inselberg Station, Nouragues, French Guiana	N 4.09412, W 52.68438
07-0080	*Mionectes macconelli*	Inselberg Station, Nouragues, French Guiana	N 4.09412, W 52.68438
07-0093	*Mionectes macconelli*	Inselberg Station, Nouragues, French Guiana	N 4.09412, W 52.68438
07-0097	*Mionectes macconelli*	Inselberg Station, Nouragues, French Guiana	N 4.09412, W 52.68438
07-0281	*Mionectes macconelli*	Inselberg Station, Nouragues, French Guiana	N 4.09412, W 52.68438
08-0868	*Mionectes macconelli*	Paracou Field Station, Paracou, French Guiana	N 5.27595, W 52.9280
08-0893	*Mionectes macconelli*	Paracou Field Station, Paracou, French Guiana	N 5.27595, W 52.9280
08-0907	*Mionectes macconelli*	Paracou Field Station, Paracou, French Guiana	N 5.27595, W 52.9280
08-0643	*Mionectes macconelli*	Parare Station, Nouragues, French Guiana	N 4.04055, W 52.6757
08-0656	*Mionectes macconelli*	Parare Station, Nouragues, French Guiana	N 4.04055, W 52.6757
08-0658	*Mionectes macconelli*	Parare Station, Nouragues, French Guiana	N 4.04055, W 52.6757
08-0712	*Mionectes macconelli*	Parare Station, Nouragues, French Guiana	N 4.04055, W 52.6757
08-0791	*Mionectes macconelli*	Parare Station, Nouragues, French Guiana	N 4.04055, W 52.6757
00N0612	*Mionectes oleagineus*	Chontacocha, Loreto, Orellana, East Ecuador	S 0.67600, W 77.25429
00N0623	*Mionectes oleagineus*	Chontacocha, Loreto, Orellana, East Ecuador	S 0.67600, W 77.25429
00N0625	*Mionectes oleagineus*	Chontacocha, Loreto, Orellana, East Ecuador	S 0.67600, W 77.25429
00N0626	*Mionectes oleagineus*	Chontacocha, Loreto, Orellana, East Ecuador	S 0.67600, W 77.25429
00N0635	*Mionectes oleagineus*	Chontacocha, Loreto, Orellana, East Ecuador	S 0.67600, W 77.25429
00N0639	*Mionectes oleagineus*	Chontacocha, Loreto, Orellana, East Ecuador	S 0.67600, W 77.25429
00N0655	*Mionectes oleagineus*	Chontacocha, Loreto, Orellana, East Ecuador	S 0.67600, W 77.25429
00N0656	*Mionectes oleagineus*	Chontacocha, Loreto, Orellana, East Ecuador	S 0.67600, W 77.25429
00N3298	*Mionectes oleagineus*	Chontacocha, Loreto, Orellana, East Ecuador	S 0.67600, W 77.25429
00N3306	*Mionectes oleagineus*	Chontacocha, Loreto, Orellana, East Ecuador	S 0.67600, W 77.25429
00N0812	*Mionectes oleagineus*	Estacion Cientifica Yasuni, Yasuni, Orellana, East Ecuador	S 0.67455, W 76.39837
00N0850	*Mionectes oleagineus*	Estacion Cientifica Yasuni, Yasuni, Orellana, East Ecuador	S 0.67455, W 76.39837
00N0861	*Mionectes oleagineus*	Estacion Cientifica Yasuni, Yasuni, Orellana, East Ecuador	S 0.67455, W 76.39837
00N0862	*Mionectes oleagineus*	Estacion Cientifica Yasuni, Yasuni, Orellana, East Ecuador	S 0.67455, W 76.39837
99N5238	*Mionectes oleagineus*	Jatun Sacha Station, Misahualli, Napo, East Ecuador	S 1.0215, W 77.62144
99N5245	*Mionectes oleagineus*	Jatun Sacha Station, Misahualli, Napo, East Ecuador	S 1.0215, W 77.62144
00N3351	*Mionectes oleagineus*	Miazal Mission, Miazal, Morona-Santiago, East Ecuador	S 2.63573, W 77.79831
00N0956	*Mionectes oleagineus*	Panacocha River, Panacocha, Sucumbios, East Ecuador	S 0.38238, W 76.17612
00N0964	*Mionectes oleagineus*	Panacocha River, Panacocha, Sucumbios, East Ecuador	S 0.38238, W 76.17612
00N0983	*Mionectes oleagineus*	Panacocha River, Panacocha, Sucumbios, East Ecuador	S 0.38238, W 76.17612
00N0986	*Mionectes oleagineus*	Panacocha River, Panacocha, Sucumbios, East Ecuador	S 0.38238, W 76.17612
00N1000	*Mionectes oleagineus*	Panacocha River, Panacocha, Sucumbios, East Ecuador	S 0.38238, W 76.17612
00N1014	*Mionectes oleagineus*	Panacocha River, Panacocha, Sucumbios, East Ecuador	S 0.38238, W 76.17612
00N1030	*Mionectes oleagineus*	Panacocha River, Panacocha, Sucumbios, East Ecuador	S 0.38238, W 76.17612

00N0702	*Mionectes oleagineus*	Tiputini Biological Station, Tiputini, Orellana, East Ecuador	S 0.63698, W 76.14912
00N0751	*Mionectes oleagineus*	Tiputini Biological Station, Tiputini, Orellana, East Ecuador	S 0.63698, W 76.14912
00N5003	*Mionectes oleagineus*	Tiputini Biological Station, Tiputini, Orellana, East Ecuador	S 0.63698, W 76.14912
00N5004	*Mionectes oleagineus*	Tiputini Biological Station, Tiputini, Orellana, East Ecuador	S 0.63698, W 76.14912
05N0055	*Mionectes oleagineus*	Tiputini Biological Station, Tiputini, Orellana, East Ecuador	S 0.63698, W 76.14912
05N0061	*Mionectes oleagineus*	Tiputini Biological Station, Tiputini, Orellana, East Ecuador	S 0.63698, W 76.14912
07-0249	*Momotus momota*	Inselberg Station, Nouragues, French Guiana	N 4.09412, W 52.68438
00N0977	*Momotus momota*	Panacocha River, Panacocha, Sucumbios, East Ecuador	S 0.38238, W 76.17612
07-0192	*Myiobius barbatus*	Inselberg Station, Nouragues, French Guiana	N 4.09412, W 52.68438
07-0199	*Myiobius barbatus*	Inselberg Station, Nouragues, French Guiana	N 4.09412, W 52.68438
07-0271	*Myiobius barbatus*	Inselberg Station, Nouragues, French Guiana	N 4.09412, W 52.68438
07-0351	*Myiobius barbatus*	Inselberg Station, Nouragues, French Guiana	N 4.09412, W 52.68438
00N3377	*Myiobius barbatus*	Miazal Mission, Miazal, Morona-Santiago, East Ecuador	S 2.63573, W 77.79831
08-0865	*Myiobius barbatus*	Paracou Field Station, Paracou, French Guiana	N 5.27595, W 52.9280
08-0797	*Myiobius barbatus*	Parare Station, Nouragues, French Guiana	N 4.04055, W 52.6757
05N0049	*Myiobius barbatus*	Tiputini Biological Station, Tiputini, Orellana, East Ecuador	S 0.63698, W 76.14912
05N0052	*Myiobius barbatus*	Tiputini Biological Station, Tiputini, Orellana, East Ecuador	S 0.63698, W 76.14912
05N0097	*Myiobius barbatus*	Tiputini Biological Station, Tiputini, Orellana, East Ecuador	S 0.63698, W 76.14912
04N5986	*Myrmotherula axillaris*	Bilsa Biological Station, Bilsa, Esmeraldas, West Ecuador	N 0.36076, W 79.71486
04N6170	*Myrmotherula axillaris*	Bilsa Biological Station, Bilsa, Esmeraldas, West Ecuador	N 0.36076, W 79.71486
04N6183	*Myrmotherula axillaris*	Bilsa Biological Station, Bilsa, Esmeraldas, West Ecuador	N 0.36076, W 79.71486
04N6414	*Myrmotherula axillaris*	Bilsa Biological Station, Bilsa, Esmeraldas, West Ecuador	N 0.36076, W 79.71486
04N8311	*Myrmotherula axillaris*	Bilsa Biological Station, Bilsa, Esmeraldas, West Ecuador	N 0.36076, W 79.71486
05N7382	*Myrmotherula axillaris*	Bilsa Biological Station, Bilsa, Esmeraldas, West Ecuador	N 0.36076, W 79.71486
05N9138	*Myrmotherula axillaris*	Bilsa Biological Station, Bilsa, Esmeraldas, West Ecuador	N 0.36076, W 79.71486
05N9532	*Myrmotherula axillaris*	Bilsa Biological Station, Bilsa, Esmeraldas, West Ecuador	N 0.36076, W 79.71486
05N9693	*Myrmotherula axillaris*	Bilsa Biological Station, Bilsa, Esmeraldas, West Ecuador	N 0.36076, W 79.71486
00N0647	*Myrmotherula axillaris*	Chontacocha, Loreto, Orellana, East Ecuador	S 0.67600, W 77.25429
00N0657	*Myrmotherula axillaris*	Chontacocha, Loreto, Orellana, East Ecuador	S 0.67600, W 77.25429
00N3282	*Myrmotherula axillaris*	Chontacocha, Loreto, Orellana, East Ecuador	S 0.67600, W 77.25429
00N3285	*Myrmotherula axillaris*	Chontacocha, Loreto, Orellana, East Ecuador	S 0.67600, W 77.25429
00N0896	*Myrmotherula axillaris*	Estacion Cientifica Yasuni, Yasuni, Orellana, East Ecuador	S 0.67455, W 76.39837
00N0597	*Myrmotherula axillaris*	Guagua Sumaco, Guagua Sumaco, Napo, East Ecuador	S 0.72493, W 77.57564
07-0142	*Myrmotherula axillaris*	Inselberg Station, Nouragues, French Guiana	N 4.09412, W 52.68438
07-0159	*Myrmotherula axillaris*	Inselberg Station, Nouragues, French Guiana	N 4.09412, W 52.68438
07-0231	*Myrmotherula axillaris*	Inselberg Station, Nouragues, French Guiana	N 4.09412, W 52.68438
07-0246	*Myrmotherula axillaris*	Inselberg Station, Nouragues, French Guiana	N 4.09412, W 52.68438
07-0324	*Myrmotherula axillaris*	Inselberg Station, Nouragues, French Guiana	N 4.09412, W 52.68438
00N3330	*Myrmotherula axillaris*	Miazal Mission, Miazal, Morona-Santiago, East Ecuador	S 2.63573, W 77.79831
00N3418	*Myrmotherula axillaris*	Miazal Mission, Miazal, Morona-Santiago, East Ecuador	S 2.63573, W 77.79831
00N3426	*Myrmotherula axillaris*	Miazal Mission, Miazal, Morona-Santiago, East Ecuador	S 2.63573, W 77.79831
00N0934	*Myrmotherula axillaris*	Panacocha River, Panacocha, Sucumbios, East Ecuador	S 0.38238, W 76.17612
00N0936	*Myrmotherula axillaris*	Panacocha River, Panacocha, Sucumbios, East Ecuador	S 0.38238, W 76.17612
00N0959	*Myrmotherula axillaris*	Panacocha River, Panacocha, Sucumbios, East Ecuador	S 0.38238, W 76.17612
00N0979	*Myrmotherula axillaris*	Panacocha River, Panacocha, Sucumbios, East Ecuador	S 0.38238, W 76.17612
00N1005	*Myrmotherula axillaris*	Panacocha River, Panacocha, Sucumbios, East Ecuador	S 0.38238, W 76.17612
08-0646	*Myrmotherula axillaris*	Parare Station, Nouragues, French Guiana	N 4.04055, W 52.6757
08-0650	*Myrmotherula axillaris*	Parare Station, Nouragues, French Guiana	N 4.04055, W 52.6757
08-0651	*Myrmotherula axillaris*	Parare Station, Nouragues, French Guiana	N 4.04055, W 52.6757
08-0707	*Myrmotherula axillaris*	Parare Station, Nouragues, French Guiana	N 4.04055, W 52.6757
08-0721	*Myrmotherula axillaris*	Parare Station, Nouragues, French Guiana	N 4.04055, W 52.6757
08-0722	*Myrmotherula axillaris*	Parare Station, Nouragues, French Guiana	N 4.04055, W 52.6757
00N0686	*Myrmotherula axillaris*	Tiputini Biological Station, Tiputini, Orellana, East Ecuador	S 0.63698, W 76.14912
00N0714	*Myrmotherula axillaris*	Tiputini Biological Station, Tiputini, Orellana, East Ecuador	S 0.63698, W 76.14912
00N1046	*Myrmotherula axillaris*	Tiputini Biological Station, Tiputini, Orellana, East Ecuador	S 0.63698, W 76.14912
00N1049	*Myrmotherula axillaris*	Tiputini Biological Station, Tiputini, Orellana, East Ecuador	S 0.63698, W 76.14912
05N0070	*Myrmotherula axillaris*	Tiputini Biological Station, Tiputini, Orellana, East Ecuador	S 0.63698, W 76.14912
05N0109	*Myrmotherula axillaris*	Tiputini Biological Station, Tiputini, Orellana, East Ecuador	S 0.63698, W 76.14912
00N0857	*Myrmotherula longipennis*	Estacion Cientifica Yasuni, Yasuni, Orellana, East Ecuador	S 0.67455, W 76.39837
07-0057	*Myrmotherula longipennis*	Inselberg Station, Nouragues, French Guiana	N 4.09412, W 52.68438
07-0277	*Myrmotherula longipennis*	Inselberg Station, Nouragues, French Guiana	N 4.09412, W 52.68438
07-0278	*Myrmotherula longipennis*	Inselberg Station, Nouragues, French Guiana	N 4.09412, W 52.68438
07-0335	*Myrmotherula longipennis*	Inselberg Station, Nouragues, French Guiana	N 4.09412, W 52.68438
07-0344	*Myrmotherula longipennis*	Inselberg Station, Nouragues, French Guiana	N 4.09412, W 52.68438
08-0895	*Myrmotherula longipennis*	Paracou Field Station, Paracou, French Guiana	N 5.27595, W 52.9280
08-0638	*Myrmotherula longipennis*	Parare Station, Nouragues, French Guiana	N 4.04055, W 52.6757
08-0673	*Myrmotherula longipennis*	Parare Station, Nouragues, French Guiana	N 4.04055, W 52.6757
08-0709	*Myrmotherula longipennis*	Parare Station, Nouragues, French Guiana	N 4.04055, W 52.6757
08-0726	*Myrmotherula longipennis*	Parare Station, Nouragues, French Guiana	N 4.04055, W 52.6757
08-0728	*Myrmotherula longipennis*	Parare Station, Nouragues, French Guiana	N 4.04055, W 52.6757
00N0703	*Myrmotherula longipennis*	Tiputini Biological Station, Tiputini, Orellana, East Ecuador	S 0.63698, W 76.14912
00N0710	*Myrmotherula longipennis*	Tiputini Biological Station, Tiputini, Orellana, East Ecuador	S 0.63698, W 76.14912
00N1047	*Myrmotherula longipennis*	Tiputini Biological Station, Tiputini, Orellana, East Ecuador	S 0.63698, W 76.14912
05N0046	*Myrmotherula longipennis*	Tiputini Biological Station, Tiputini, Orellana, East Ecuador	S 0.63698, W 76.14912
05N0072	*Myrmotherula longipennis*	Tiputini Biological Station, Tiputini, Orellana, East Ecuador	S 0.63698, W 76.14912
07-0189	*Myrmotherula menetriesii*	Inselberg Station, Nouragues, French Guiana	N 4.09412, W 52.68438
07-0193	*Myrmotherula menetriesii*	Inselberg Station, Nouragues, French Guiana	N 4.09412, W 52.68438
07-0331	*Myrmotherula menetriesii*	Inselberg Station, Nouragues, French Guiana	N 4.09412, W 52.68438
00N3321	*Myrmotherula menetriesii*	Miazal Mission, Miazal, Morona-Santiago, East Ecuador	S 2.63573, W 77.79831
00N3356	*Myrmotherula menetriesii*	Miazal Mission, Miazal, Morona-Santiago, East Ecuador	S 2.63573, W 77.79831
00N3421	*Myrmotherula menetriesii*	Miazal Mission, Miazal, Morona-Santiago, East Ecuador	S 2.63573, W 77.79831
08-0750	*Myrmotherula menetriesii*	Parare Station, Nouragues, French Guiana	N 4.04055, W 52.6757
00N0718	*Myrmotherula menetriesii*	Tiputini Biological Station, Tiputini, Orellana, East Ecuador	S 0.63698, W 76.14912
00N5028	*Myrmotherula menetriesii*	Tiputini Biological Station, Tiputini, Orellana, East Ecuador	S 0.63698, W 76.14912
05N0071	*Myrmotherula menetriesii*	Tiputini Biological Station, Tiputini, Orellana, East Ecuador	S 0.63698, W 76.14912
00N0801	*Phaetornis bourcieri*	Estacion Cientifica Yasuni, Yasuni, Orellana, East Ecuador	S 0.67455, W 76.39837
00N0813	*Phaetornis bourcieri*	Estacion Cientifica Yasuni, Yasuni, Orellana, East Ecuador	S 0.67455, W 76.39837
07-0034	*Phaetornis bourcieri*	Inselberg Station, Nouragues, French Guiana	N 4.09412, W 52.68438
07-0118	*Phaetornis bourcieri*	Inselberg Station, Nouragues, French Guiana	N 4.09412, W 52.68438
07-0127	*Phaetornis bourcieri*	Inselberg Station, Nouragues, French Guiana	N 4.09412, W 52.68438
07-0137	*Phaetornis bourcieri*	Inselberg Station, Nouragues, French Guiana	N 4.09412, W 52.68438
07-0190	*Phaetornis bourcieri*	Inselberg Station, Nouragues, French Guiana	N 4.09412, W 52.68438

07-0220	*Phaetornis bourcieri*	Inselberg Station, Nouragues, French Guiana	N 4.09412, W 52.68438
07-0241	*Phaetornis bourcieri*	Inselberg Station, Nouragues, French Guiana	N 4.09412, W 52.68438
99N5249	*Phaetornis bourcieri*	Jatun Sacha Station, Misahualli, Napo, East Ecuador	S 1.0215, W 77.62144
00N1001	*Phaetornis bourcieri*	Panacocha River, Panacocha, Sucumbios, East Ecuador	S 0.38238, W 76.17612
00N1026	*Phaetornis bourcieri*	Panacocha River, Panacocha, Sucumbios, East Ecuador	S 0.38238, W 76.17612
00N0723	*Phaetornis bourcieri*	Tiputini Biological Station, Tiputini, Orellana, East Ecuador	S 0.63698, W 76.14912
00N0741	*Phaetornis bourcieri*	Tiputini Biological Station, Tiputini, Orellana, East Ecuador	S 0.63698, W 76.14912
07-0029	*Philydor erythrocercum*	Inselberg Station, Nouragues, French Guiana	N 4.09412, W 52.68438
07-0085	*Philydor erythrocercum*	Inselberg Station, Nouragues, French Guiana	N 4.09412, W 52.68438
07-0286	*Philydor erythrocercum*	Inselberg Station, Nouragues, French Guiana	N 4.09412, W 52.68438
07-0343	*Philydor erythrocercum*	Inselberg Station, Nouragues, French Guiana	N 4.09412, W 52.68438
08-0715	*Philydor erythrocercum*	Parare Station, Nouragues, French Guiana	N 4.04055, W 52.6757
08-0727	*Philydor erythrocercum*	Parare Station, Nouragues, French Guiana	N 4.04055, W 52.6757
05N0104	*Philydor erythrocercum*	Tiputini Biological Station, Tiputini, Orellana, East Ecuador	S 0.63698, W 76.14912
05N0108	*Philydor erythrocercum*	Tiputini Biological Station, Tiputini, Orellana, East Ecuador	S 0.63698, W 76.14912
04N8309	*Pipra erythrocephala*	Bilsa Biological Station, Bilsa, Esmeraldas, West Ecuador	N 0.36076, W 79.71486
04N8462	*Pipra erythrocephala*	Bilsa Biological Station, Bilsa, Esmeraldas, West Ecuador	N 0.36076, W 79.71486
05N8559	*Pipra erythrocephala*	Bilsa Biological Station, Bilsa, Esmeraldas, West Ecuador	N 0.36076, W 79.71486
05N9545	*Pipra erythrocephala*	Bilsa Biological Station, Bilsa, Esmeraldas, West Ecuador	N 0.36076, W 79.71486
06N1737	*Pipra erythrocephala*	Bilsa Biological Station, Bilsa, Esmeraldas, West Ecuador	N 0.36076, W 79.71486
00N0622	*Pipra erythrocephala*	Chontacocha, Loreto, Orellana, East Ecuador	S 0.67600, W 77.25429
00N0817	*Pipra erythrocephala*	Estacion Cientifica Yasuni, Yasuni, Orellana, East Ecuador	S 0.67455, W 76.39837
00N0852	*Pipra erythrocephala*	Estacion Cientifica Yasuni, Yasuni, Orellana, East Ecuador	S 0.67455, W 76.39837
07-0178	*Pipra erythrocephala*	Inselberg Station, Nouragues, French Guiana	N 4.09412, W 52.68438
07-0244	*Pipra erythrocephala*	Inselberg Station, Nouragues, French Guiana	N 4.09412, W 52.68438
00N3311	*Pipra erythrocephala*	Miazal Mission, Miazal, Morona-Santiago, East Ecuador	S 2.63573, W 77.79831
00N3320	*Pipra erythrocephala*	Miazal Mission, Miazal, Morona-Santiago, East Ecuador	S 2.63573, W 77.79831
00N3347	*Pipra erythrocephala*	Miazal Mission, Miazal, Morona-Santiago, East Ecuador	S 2.63573, W 77.79831
00N3354	*Pipra erythrocephala*	Miazal Mission, Miazal, Morona-Santiago, East Ecuador	S 2.63573, W 77.79831
00N3359	*Pipra erythrocephala*	Miazal Mission, Miazal, Morona-Santiago, East Ecuador	S 2.63573, W 77.79831
00N3361	*Pipra erythrocephala*	Miazal Mission, Miazal, Morona-Santiago, East Ecuador	S 2.63573, W 77.79831
00N3364	*Pipra erythrocephala*	Miazal Mission, Miazal, Morona-Santiago, East Ecuador	S 2.63573, W 77.79831
00N3383	*Pipra erythrocephala*	Miazal Mission, Miazal, Morona-Santiago, East Ecuador	S 2.63573, W 77.79831
00N3441	*Pipra erythrocephala*	Miazal Mission, Miazal, Morona-Santiago, East Ecuador	S 2.63573, W 77.79831
00N0961	*Pipra erythrocephala*	Panacocha River, Panacocha, Sucumbios, East Ecuador	S 0.38238, W 76.17612
00N0978	*Pipra erythrocephala*	Panacocha River, Panacocha, Sucumbios, East Ecuador	S 0.38238, W 76.17612
00N1023	*Pipra erythrocephala*	Panacocha River, Panacocha, Sucumbios, East Ecuador	S 0.38238, W 76.17612
08-0621	*Pipra erythrocephala*	Parare Station, Nouragues, French Guiana	N 4.04055, W 52.6757
08-0648	*Pipra erythrocephala*	Parare Station, Nouragues, French Guiana	N 4.04055, W 52.6757
08-0649	*Pipra erythrocephala*	Parare Station, Nouragues, French Guiana	N 4.04055, W 52.6757
08-0804	*Pipra erythrocephala*	Parare Station, Nouragues, French Guiana	N 4.04055, W 52.6757
00N0783	*Pipra erythrocephala*	Tiputini Biological Station, Tiputini, Orellana, East Ecuador	S 0.63698, W 76.14912
00N5023	*Pipra erythrocephala*	Tiputini Biological Station, Tiputini, Orellana, East Ecuador	S 0.63698, W 76.14912
00N0608	*Pithys albifrons*	Chontacocha, Loreto, Orellana, East Ecuador	S 0.67600, W 77.25429
02N9199	*Pithys albifrons*	Cumanda, Cumanda, Pastaza, East Ecuador	S 1.47686, W 78.14325
02N9229	*Pithys albifrons*	Cumanda, Cumanda, Pastaza, East Ecuador	S 1.47686, W 78.14325
00N0796	*Pithys albifrons*	Estacion Cientifica Yasuni, Yasuni, Orellana, East Ecuador	S 0.67455, W 76.39837
00N0797	*Pithys albifrons*	Estacion Cientifica Yasuni, Yasuni, Orellana, East Ecuador	S 0.67455, W 76.39837
00N0805	*Pithys albifrons*	Estacion Cientifica Yasuni, Yasuni, Orellana, East Ecuador	S 0.67455, W 76.39837
00N0893	*Pithys albifrons*	Estacion Cientifica Yasuni, Yasuni, Orellana, East Ecuador	S 0.67455, W 76.39837
00N0894	*Pithys albifrons*	Estacion Cientifica Yasuni, Yasuni, Orellana, East Ecuador	S 0.67455, W 76.39837
07-0002	*Pithys albifrons*	Inselberg Station, Nouragues, French Guiana	N 4.09412, W 52.68438
07-0012	*Pithys albifrons*	Inselberg Station, Nouragues, French Guiana	N 4.09412, W 52.68438
07-0022	*Pithys albifrons*	Inselberg Station, Nouragues, French Guiana	N 4.09412, W 52.68438
07-0040	*Pithys albifrons*	Inselberg Station, Nouragues, French Guiana	N 4.09412, W 52.68438
07-0043	*Pithys albifrons*	Inselberg Station, Nouragues, French Guiana	N 4.09412, W 52.68438
07-0124	*Pithys albifrons*	Inselberg Station, Nouragues, French Guiana	N 4.09412, W 52.68438
07-0143	*Pithys albifrons*	Inselberg Station, Nouragues, French Guiana	N 4.09412, W 52.68438
07-0188	*Pithys albifrons*	Inselberg Station, Nouragues, French Guiana	N 4.09412, W 52.68438
07-0207	*Pithys albifrons*	Inselberg Station, Nouragues, French Guiana	N 4.09412, W 52.68438
07-0210	*Pithys albifrons*	Inselberg Station, Nouragues, French Guiana	N 4.09412, W 52.68438
07-0256	*Pithys albifrons*	Inselberg Station, Nouragues, French Guiana	N 4.09412, W 52.68438
07-0282	*Pithys albifrons*	Inselberg Station, Nouragues, French Guiana	N 4.09412, W 52.68438
07-0319	*Pithys albifrons*	Inselberg Station, Nouragues, French Guiana	N 4.09412, W 52.68438
07-0322	*Pithys albifrons*	Inselberg Station, Nouragues, French Guiana	N 4.09412, W 52.68438
99N5253	*Pithys albifrons*	Jatun Sacha Station, Misahualli, Napo, East Ecuador	S 1.0215, W 77.62144
00N3397	*Pithys albifrons*	Miazal Mission, Miazal, Morona-Santiago, East Ecuador	S 2.63573, W 77.79831
08-0817	*Pithys albifrons*	Paracou Field Station, Paracou, French Guiana	N 5.27595, W 52.9280
08-0821	*Pithys albifrons*	Paracou Field Station, Paracou, French Guiana	N 5.27595, W 52.9280
08-0825	*Pithys albifrons*	Paracou Field Station, Paracou, French Guiana	N 5.27595, W 52.9280
08-0826	*Pithys albifrons*	Paracou Field Station, Paracou, French Guiana	N 5.27595, W 52.9280
08-0846	*Pithys albifrons*	Paracou Field Station, Paracou, French Guiana	N 5.27595, W 52.9280
08-0782	*Pithys albifrons*	Parare Station, Nouragues, French Guiana	N 4.04055, W 52.6757
08-0786	*Pithys albifrons*	Parare Station, Nouragues, French Guiana	N 4.04055, W 52.6757
00N0695	*Pithys albifrons*	Tiputini Biological Station, Tiputini, Orellana, East Ecuador	S 0.63698, W 76.14912
00N0754	*Pithys albifrons*	Tiputini Biological Station, Tiputini, Orellana, East Ecuador	S 0.63698, W 76.14912
00N0781	*Pithys albifrons*	Tiputini Biological Station, Tiputini, Orellana, East Ecuador	S 0.63698, W 76.14912
00N5002	*Pithys albifrons*	Tiputini Biological Station, Tiputini, Orellana, East Ecuador	S 0.63698, W 76.14912
00N5029	*Pithys albifrons*	Tiputini Biological Station, Tiputini, Orellana, East Ecuador	S 0.63698, W 76.14912
00N5033	*Pithys albifrons*	Tiputini Biological Station, Tiputini, Orellana, East Ecuador	S 0.63698, W 76.14912
00N5035	*Pithys albifrons*	Tiputini Biological Station, Tiputini, Orellana, East Ecuador	S 0.63698, W 76.14912
00N5042	*Pithys albifrons*	Tiputini Biological Station, Tiputini, Orellana, East Ecuador	S 0.63698, W 76.14912
00N5064	*Pithys albifrons*	Tiputini Biological Station, Tiputini, Orellana, East Ecuador	S 0.63698, W 76.14912
00N5072	*Pithys albifrons*	Tiputini Biological Station, Tiputini, Orellana, East Ecuador	S 0.63698, W 76.14912
05N0038	*Pithys albifrons*	Tiputini Biological Station, Tiputini, Orellana, East Ecuador	S 0.63698, W 76.14912
05N0067	*Pithys albifrons*	Tiputini Biological Station, Tiputini, Orellana, East Ecuador	S 0.63698, W 76.14912
05N0069	*Pithys albifrons*	Tiputini Biological Station, Tiputini, Orellana, East Ecuador	S 0.63698, W 76.14912
05N0087	*Pithys albifrons*	Tiputini Biological Station, Tiputini, Orellana, East Ecuador	S 0.63698, W 76.14912
04N5612	*Platyrinchus coronatus*	Bilsa Biological Station, Bilsa, Esmeraldas, West Ecuador	N 0.36076, W 79.71486
04N5633	*Platyrinchus coronatus*	Bilsa Biological Station, Bilsa, Esmeraldas, West Ecuador	N 0.36076, W 79.71486
04N6277	*Platyrinchus coronatus*	Bilsa Biological Station, Bilsa, Esmeraldas, West Ecuador	N 0.36076, W 79.71486
04N8317	*Platyrinchus coronatus*	Bilsa Biological Station, Bilsa, Esmeraldas, West Ecuador	N 0.36076, W 79.71486

04N8482	*Platyrinchus coronatus*	Bilsa Biological Station, Bilsa, Esmeraldas, West Ecuador	N 0.36076, W 79.71486
05N7327	*Platyrinchus coronatus*	Bilsa Biological Station, Bilsa, Esmeraldas, West Ecuador	N 0.36076, W 79.71486
05N7456	*Platyrinchus coronatus*	Bilsa Biological Station, Bilsa, Esmeraldas, West Ecuador	N 0.36076, W 79.71486
00N0637	*Platyrinchus coronatus*	Chontacocha, Loreto, Orellana, East Ecuador	S 0.67600, W 77.25429
07-0010	*Platyrinchus coronatus*	Inselberg Station, Nouragues, French Guiana	N 4.09412, W 52.68438
07-0100	*Platyrinchus coronatus*	Inselberg Station, Nouragues, French Guiana	N 4.09412, W 52.68438
07-0221	*Platyrinchus coronatus*	Inselberg Station, Nouragues, French Guiana	N 4.09412, W 52.68438
07-0222	*Platyrinchus coronatus*	Inselberg Station, Nouragues, French Guiana	N 4.09412, W 52.68438
07-0223	*Platyrinchus coronatus*	Inselberg Station, Nouragues, French Guiana	N 4.09412, W 52.68438
08-0659	*Platyrinchus coronatus*	Parare Station, Nouragues, French Guiana	N 4.04055, W 52.6757
08-0686	*Platyrinchus coronatus*	Parare Station, Nouragues, French Guiana	N 4.04055, W 52.6757
08-0737	*Platyrinchus coronatus*	Parare Station, Nouragues, French Guiana	N 4.04055, W 52.6757
08-0740	*Platyrinchus coronatus*	Parare Station, Nouragues, French Guiana	N 4.04055, W 52.6757
08-0793	*Platyrinchus coronatus*	Parare Station, Nouragues, French Guiana	N 4.04055, W 52.6757
00N0662	*Platyrinchus coronatus*	Tiputini Biological Station, Tiputini, Orellana, East Ecuador	S 0.63698, W 76.14912
00N0663	*Platyrinchus coronatus*	Tiputini Biological Station, Tiputini, Orellana, East Ecuador	S 0.63698, W 76.14912
00N0675	*Platyrinchus coronatus*	Tiputini Biological Station, Tiputini, Orellana, East Ecuador	S 0.63698, W 76.14912
00N0733	*Platyrinchus coronatus*	Tiputini Biological Station, Tiputini, Orellana, East Ecuador	S 0.63698, W 76.14912
00N0749	*Platyrinchus coronatus*	Tiputini Biological Station, Tiputini, Orellana, East Ecuador	S 0.63698, W 76.14912
05N0041	*Platyrinchus coronatus*	Tiputini Biological Station, Tiputini, Orellana, East Ecuador	S 0.63698, W 76.14912
00N0846	*Rhynchocyclus olivaceus*	Estacion Cientifica Yasuni, Yasuni, Orellana, East Ecuador	S 0.67455, W 76.39837
07-0327	*Rhynchocyclus olivaceus*	Inselberg Station, Nouragues, French Guiana	N 4.09412, W 52.68438
00N3437	*Rhynchocyclus olivaceus*	Miazal Mission, Miazal, Morona-Santiago, East Ecuador	S 2.63573, W 77.79831
08-0857	*Rhynchocyclus olivaceus*	Paracou Field Station, Paracou, French Guiana	N 5.27595, W 52.9280
08-0723	*Rhynchocyclus olivaceus*	Parare Station, Nouragues, French Guiana	N 4.04055, W 52.6757
08-0766	*Rhynchocyclus olivaceus*	Parare Station, Nouragues, French Guiana	N 4.04055, W 52.6757
04N6080	*Schiffornis turdina*	Bilsa Biological Station, Bilsa, Esmeraldas, West Ecuador	N 0.36076, W 79.71486
04N6083	*Schiffornis turdina*	Bilsa Biological Station, Bilsa, Esmeraldas, West Ecuador	N 0.36076, W 79.71486
04N6281	*Schiffornis turdina*	Bilsa Biological Station, Bilsa, Esmeraldas, West Ecuador	N 0.36076, W 79.71486
04N6764	*Schiffornis turdina*	Bilsa Biological Station, Bilsa, Esmeraldas, West Ecuador	N 0.36076, W 79.71486
04N7915	*Schiffornis turdina*	Bilsa Biological Station, Bilsa, Esmeraldas, West Ecuador	N 0.36076, W 79.71486
00N0564	*Schiffornis turdina*	Guagua Sumaco, Guagua Sumaco, Napo, East Ecuador	S 0.72493, W 77.57564
00N3171	*Schiffornis turdina*	Hollin River, Hollin, Napo, East Ecuador	S 0.68896, W 77.72658
02N9114	*Schiffornis turdina*	Hollin River, Hollin, Napo, East Ecuador	S 0.68896, W 77.72658
07-0071	*Schiffornis turdina*	Inselberg Station, Nouragues, French Guiana	N 4.09412, W 52.68438
07-0147	*Schiffornis turdina*	Inselberg Station, Nouragues, French Guiana	N 4.09412, W 52.68438
07-0264	*Schiffornis turdina*	Inselberg Station, Nouragues, French Guiana	N 4.09412, W 52.68438
07-0311	*Schiffornis turdina*	Inselberg Station, Nouragues, French Guiana	N 4.09412, W 52.68438
00N3526	*Schiffornis turdina*	Nueva Alianza, Sangay National Park, Morona-Santiago, East Ecuador	S 2.09883, W 78.15164
00N5015	*Schiffornis turdina*	Tiputini Biological Station, Tiputini, Orellana, East Ecuador	S 0.63698, W 76.14912
05N7441	*Sclerurus mexicanus*	Bilsa Biological Station, Bilsa, Esmeraldas, West Ecuador	N 0.36076, W 79.71486
05N8997	*Sclerurus mexicanus*	Bilsa Biological Station, Bilsa, Esmeraldas, West Ecuador	N 0.36076, W 79.71486
05N9053	*Sclerurus mexicanus*	Bilsa Biological Station, Bilsa, Esmeraldas, West Ecuador	N 0.36076, W 79.71486
00N3231	*Sclerurus mexicanus*	Hollin River, Hollin, Napo, East Ecuador	S 0.68896, W 77.72658
07-0087	*Sclerurus mexicanus*	Inselberg Station, Nouragues, French Guiana	N 4.09412, W 52.68438
00N3535	*Sclerurus mexicanus*	Nueva Alianza, Sangay National Park, Morona-Santiago, East Ecuador	S 2.09883, W 78.15164
00N3542	*Sclerurus mexicanus*	Nueva Alianza, Sangay National Park, Morona-Santiago, East Ecuador	S 2.09883, W 78.15164
02N9350	*Sclerurus mexicanus*	Nueva Alianza, Sangay National Park, Morona-Santiago, East Ecuador	S 2.09883, W 78.15164
00N5049	*Sclerurus mexicanus*	Tiputini Biological Station, Tiputini, Orellana, East Ecuador	S 0.63698, W 76.14912
00N5054	*Sclerurus mexicanus*	Tiputini Biological Station, Tiputini, Orellana, East Ecuador	S 0.63698, W 76.14912
00N3278	*Tachyphonus surinamus*	Chontacocha, Loreto, Orellana, East Ecuador	S 0.67600, W 77.25429
00N3281	*Tachyphonus surinamus*	Chontacocha, Loreto, Orellana, East Ecuador	S 0.67600, W 77.25429
00N0888	*Tachyphonus surinamus*	Estacion Cientifica Yasuni, Yasuni, Orellana, East Ecuador	S 0.67455, W 76.39837
07-0185	*Tachyphonus surinamus*	Inselberg Station, Nouragues, French Guiana	N 4.09412, W 52.68438
07-0196	*Tachyphonus surinamus*	Inselberg Station, Nouragues, French Guiana	N 4.09412, W 52.68438
07-0202	*Tachyphonus surinamus*	Inselberg Station, Nouragues, French Guiana	N 4.09412, W 52.68438
07-0213	*Tachyphonus surinamus*	Inselberg Station, Nouragues, French Guiana	N 4.09412, W 52.68438
07-0216	*Tachyphonus surinamus*	Inselberg Station, Nouragues, French Guiana	N 4.09412, W 52.68438
07-0217	*Tachyphonus surinamus*	Inselberg Station, Nouragues, French Guiana	N 4.09412, W 52.68438
07-0218	*Tachyphonus surinamus*	Inselberg Station, Nouragues, French Guiana	N 4.09412, W 52.68438
07-0219	*Tachyphonus surinamus*	Inselberg Station, Nouragues, French Guiana	N 4.09412, W 52.68438
00N3314	*Tachyphonus surinamus*	Miazal Mission, Miazal, Morona-Santiago, East Ecuador	S 2.63573, W 77.79831
00N3438	*Tachyphonus surinamus*	Miazal Mission, Miazal, Morona-Santiago, East Ecuador	S 2.63573, W 77.79831
08-0892	*Tachyphonus surinamus*	Paracou Field Station, Paracou, French Guiana	N 5.27595, W 52.9280
04N0471	*Thalurania fannyi*	Los Bancos, Los Bancos, Pichincha, West Ecuador	N 0.06479, W 78.98241
04N0472	*Thalurania fannyi*	Los Bancos, Los Bancos, Pichincha, West Ecuador	N 0.06479, W 78.98241
04N0335	*Thalurania fannyi*	Reserva Forestal La Magusa, Las Delicias, Pichincha, West Ecuador	N 0.16386, W 78.86964
04N0384	*Thalurania fannyi*	Reserva Forestal La Magusa, Las Delicias, Pichincha, West Ecuador	N 0.16386, W 78.86964
04N0391	*Thalurania fannyi*	Reserva Forestal La Magusa, Las Delicias, Pichincha, West Ecuador	N 0.16386, W 78.86964
04N0580	*Thalurania fannyi*	Reserva Forestal La Magusa, Las Delicias, Pichincha, West Ecuador	N 0.16386, W 78.86964
00N0654	*Thalurania furcata*	Chontacocha, Loreto, Orellana, East Ecuador	S 0.67600, W 77.25429
00N0501	*Thalurania furcata*	Hollin River, Hollin, Napo, East Ecuador	S 0.68896, W 77.72658
02N9092	*Thalurania furcata*	Hollin River, Hollin, Napo, East Ecuador	S 0.68896, W 77.72658
02N9143	*Thalurania furcata*	Hollin River, Hollin, Napo, East Ecuador	S 0.68896, W 77.72658
07-0052	*Thalurania furcata*	Inselberg Station, Nouragues, French Guiana	N 4.09412, W 52.68438
07-0065	*Thalurania furcata*	Inselberg Station, Nouragues, French Guiana	N 4.09412, W 52.68438
07-0067	*Thalurania furcata*	Inselberg Station, Nouragues, French Guiana	N 4.09412, W 52.68438
07-0081	*Thalurania furcata*	Inselberg Station, Nouragues, French Guiana	N 4.09412, W 52.68438
07-0082	*Thalurania furcata*	Inselberg Station, Nouragues, French Guiana	N 4.09412, W 52.68438
07-0149	*Thalurania furcata*	Inselberg Station, Nouragues, French Guiana	N 4.09412, W 52.68438
07-0150	*Thalurania furcata*	Inselberg Station, Nouragues, French Guiana	N 4.09412, W 52.68438
07-0168	*Thalurania furcata*	Inselberg Station, Nouragues, French Guiana	N 4.09412, W 52.68438
07-0170	*Thalurania furcata*	Inselberg Station, Nouragues, French Guiana	N 4.09412, W 52.68438
00N3362	*Thalurania furcata*	Miazal Mission, Miazal, Morona-Santiago, East Ecuador	S 2.63573, W 77.79831
00N3366	*Thalurania furcata*	Miazal Mission, Miazal, Morona-Santiago, East Ecuador	S 2.63573, W 77.79831
00N3424	*Thalurania furcata*	Miazal Mission, Miazal, Morona-Santiago, East Ecuador	S 2.63573, W 77.79831
00N3429	*Thalurania furcata*	Miazal Mission, Miazal, Morona-Santiago, East Ecuador	S 2.63573, W 77.79831
00N3491	*Thalurania furcata*	Nueva Alianza, Sangay National Park, Morona-Santiago, East Ecuador	S 2.09883, W 78.15164
00N3507	*Thalurania furcata*	Nueva Alianza, Sangay National Park, Morona-Santiago, East Ecuador	S 2.09883, W 78.15164
08-0706	*Thalurania furcata*	Parare Station, Nouragues, French Guiana	N 4.04055, W 52.6757
00N0722	*Thalurania furcata*	Tiputini Biological Station, Tiputini, Orellana, East Ecuador	S 0.63698, W 76.14912
00N0734	*Thalurania furcata*	Tiputini Biological Station, Tiputini, Orellana, East Ecuador	S 0.63698, W 76.14912

00N5062	*Thalurania furcata*	Tiputini Biological Station, Tiputini, Orellana, East Ecuador	S 0.63698, W 76.14912
00N3253	*Thamnomanes ardesiacus*	Chontacocha, Loreto, Orellana, East Ecuador	S 0.67600, W 77.25429
00N3265	*Thamnomanes ardesiacus*	Chontacocha, Loreto, Orellana, East Ecuador	S 0.67600, W 77.25429
00N0823	*Thamnomanes ardesiacus*	Estacion Cientifica Yasuni, Yasuni, Orellana, East Ecuador	S 0.67455, W 76.39837
00N0826	*Thamnomanes ardesiacus*	Estacion Cientifica Yasuni, Yasuni, Orellana, East Ecuador	S 0.67455, W 76.39837
07-0006	*Thamnomanes ardesiacus*	Inselberg Station, Nouragues, French Guiana	N 4.09412, W 52.68438
07-0086	*Thamnomanes ardesiacus*	Inselberg Station, Nouragues, French Guiana	N 4.09412, W 52.68438
07-0145	*Thamnomanes ardesiacus*	Inselberg Station, Nouragues, French Guiana	N 4.09412, W 52.68438
07-0177	*Thamnomanes ardesiacus*	Inselberg Station, Nouragues, French Guiana	N 4.09412, W 52.68438
07-0182	*Thamnomanes ardesiacus*	Inselberg Station, Nouragues, French Guiana	N 4.09412, W 52.68438
07-0238	*Thamnomanes ardesiacus*	Inselberg Station, Nouragues, French Guiana	N 4.09412, W 52.68438
07-0250	*Thamnomanes ardesiacus*	Inselberg Station, Nouragues, French Guiana	N 4.09412, W 52.68438
07-0254	*Thamnomanes ardesiacus*	Inselberg Station, Nouragues, French Guiana	N 4.09412, W 52.68438
07-0269	*Thamnomanes ardesiacus*	Inselberg Station, Nouragues, French Guiana	N 4.09412, W 52.68438
07-0273	*Thamnomanes ardesiacus*	Inselberg Station, Nouragues, French Guiana	N 4.09412, W 52.68438
07-0274	*Thamnomanes ardesiacus*	Inselberg Station, Nouragues, French Guiana	N 4.09412, W 52.68438
07-0328	*Thamnomanes ardesiacus*	Inselberg Station, Nouragues, French Guiana	N 4.09412, W 52.68438
07-0358	*Thamnomanes ardesiacus*	Inselberg Station, Nouragues, French Guiana	N 4.09412, W 52.68438
00N0908	*Thamnomanes ardesiacus*	Panacocha River, Panacocha, Sucumbios, East Ecuador	S 0.38238, W 76.17612
00N0912	*Thamnomanes ardesiacus*	Panacocha River, Panacocha, Sucumbios, East Ecuador	S 0.38238, W 76.17612
00N0913	*Thamnomanes ardesiacus*	Panacocha River, Panacocha, Sucumbios, East Ecuador	S 0.38238, W 76.17612
00N0935	*Thamnomanes ardesiacus*	Panacocha River, Panacocha, Sucumbios, East Ecuador	S 0.38238, W 76.17612
00N0968	*Thamnomanes ardesiacus*	Panacocha River, Panacocha, Sucumbios, East Ecuador	S 0.38238, W 76.17612
00N0985	*Thamnomanes ardesiacus*	Panacocha River, Panacocha, Sucumbios, East Ecuador	S 0.38238, W 76.17612
00N1004	*Thamnomanes ardesiacus*	Panacocha River, Panacocha, Sucumbios, East Ecuador	S 0.38238, W 76.17612
00N1006	*Thamnomanes ardesiacus*	Panacocha River, Panacocha, Sucumbios, East Ecuador	S 0.38238, W 76.17612
00N1022	*Thamnomanes ardesiacus*	Panacocha River, Panacocha, Sucumbios, East Ecuador	S 0.38238, W 76.17612
08-0860	*Thamnomanes ardesiacus*	Paracou Field Station, Paracou, French Guiana	N 5.27595, W 52.9280
08-0862	*Thamnomanes ardesiacus*	Paracou Field Station, Paracou, French Guiana	N 5.27595, W 52.9280
08-0872	*Thamnomanes ardesiacus*	Paracou Field Station, Paracou, French Guiana	N 5.27595, W 52.9280
08-0694	*Thamnomanes ardesiacus*	Parare Station, Nouragues, French Guiana	N 4.04055, W 52.6757
08-0713	*Thamnomanes ardesiacus*	Parare Station, Nouragues, French Guiana	N 4.04055, W 52.6757
08-0720	*Thamnomanes ardesiacus*	Parare Station, Nouragues, French Guiana	N 4.04055, W 52.6757
08-0725	*Thamnomanes ardesiacus*	Parare Station, Nouragues, French Guiana	N 4.04055, W 52.6757
08-0769	*Thamnomanes ardesiacus*	Parare Station, Nouragues, French Guiana	N 4.04055, W 52.6757
00N0661	*Thamnomanes ardesiacus*	Tiputini Biological Station, Tiputini, Orellana, East Ecuador	S 0.63698, W 76.14912
00N0673	*Thamnomanes ardesiacus*	Tiputini Biological Station, Tiputini, Orellana, East Ecuador	S 0.63698, W 76.14912
00N0725	*Thamnomanes ardesiacus*	Tiputini Biological Station, Tiputini, Orellana, East Ecuador	S 0.63698, W 76.14912
00N0772	*Thamnomanes ardesiacus*	Tiputini Biological Station, Tiputini, Orellana, East Ecuador	S 0.63698, W 76.14912
00N0780	*Thamnomanes ardesiacus*	Tiputini Biological Station, Tiputini, Orellana, East Ecuador	S 0.63698, W 76.14912
00N0782	*Thamnomanes ardesiacus*	Tiputini Biological Station, Tiputini, Orellana, East Ecuador	S 0.63698, W 76.14912
00N1050	*Thamnomanes ardesiacus*	Tiputini Biological Station, Tiputini, Orellana, East Ecuador	S 0.63698, W 76.14912
00N5011	*Thamnomanes ardesiacus*	Tiputini Biological Station, Tiputini, Orellana, East Ecuador	S 0.63698, W 76.14912
00N5039	*Thamnomanes ardesiacus*	Tiputini Biological Station, Tiputini, Orellana, East Ecuador	S 0.63698, W 76.14912
05N0009	*Thamnomanes ardesiacus*	Tiputini Biological Station, Tiputini, Orellana, East Ecuador	S 0.63698, W 76.14912
05N0023	*Thamnomanes ardesiacus*	Tiputini Biological Station, Tiputini, Orellana, East Ecuador	S 0.63698, W 76.14912
05N0026	*Thamnomanes ardesiacus*	Tiputini Biological Station, Tiputini, Orellana, East Ecuador	S 0.63698, W 76.14912
05N0063	*Thamnomanes ardesiacus*	Tiputini Biological Station, Tiputini, Orellana, East Ecuador	S 0.63698, W 76.14912
07-0112	*Thamnomanes caesius*	Inselberg Station, Nouragues, French Guiana	N 4.09412, W 52.68438
07-0326	*Thamnomanes caesius*	Inselberg Station, Nouragues, French Guiana	N 4.09412, W 52.68438
00N3376	*Thamnomanes caesius*	Miazal Mission, Miazal, Morona-Santiago, East Ecuador	S 2.63573, W 77.79831
08-0905	*Thamnomanes caesius*	Paracou Field Station, Paracou, French Guiana	N 5.27595, W 52.9280
08-0714	*Thamnomanes caesius*	Parare Station, Nouragues, French Guiana	N 4.04055, W 52.6757
08-0717	*Thamnomanes caesius*	Parare Station, Nouragues, French Guiana	N 4.04055, W 52.6757
00N0659	*Thamnomanes caesius*	Tiputini Biological Station, Tiputini, Orellana, East Ecuador	S 0.63698, W 76.14912
00N0706	*Thamnomanes caesius*	Tiputini Biological Station, Tiputini, Orellana, East Ecuador	S 0.63698, W 76.14912
00N0707	*Thamnomanes caesius*	Tiputini Biological Station, Tiputini, Orellana, East Ecuador	S 0.63698, W 76.14912
00N0743	*Thamnomanes caesius*	Tiputini Biological Station, Tiputini, Orellana, East Ecuador	S 0.63698, W 76.14912
00N5000	*Thamnomanes caesius*	Tiputini Biological Station, Tiputini, Orellana, East Ecuador	S 0.63698, W 76.14912
00N5040	*Thamnomanes caesius*	Tiputini Biological Station, Tiputini, Orellana, East Ecuador	S 0.63698, W 76.14912
05N0112	*Thamnomanes caesius*	Tiputini Biological Station, Tiputini, Orellana, East Ecuador	S 0.63698, W 76.14912
04N6509	*Trogon rufus*	Bilsa Biological Station, Bilsa, Esmeraldas, West Ecuador	N 0.36076, W 79.71486
05N8688	*Trogon rufus*	Bilsa Biological Station, Bilsa, Esmeraldas, West Ecuador	N 0.36076, W 79.71486
05N8962	*Trogon rufus*	Bilsa Biological Station, Bilsa, Esmeraldas, West Ecuador	N 0.36076, W 79.71486
05N9841	*Trogon rufus*	Bilsa Biological Station, Bilsa, Esmeraldas, West Ecuador	N 0.36076, W 79.71486
07-0295	*Trogon rufus*	Inselberg Station, Nouragues, French Guiana	N 4.09412, W 52.68438
00N0927	*Trogon rufus*	Panacocha River, Panacocha, Sucumbios, East Ecuador	S 0.38238, W 76.17612
00N0738	*Trogon rufus*	Tiputini Biological Station, Tiputini, Orellana, East Ecuador	S 0.63698, W 76.14912
02N9232	*Turdus albicollis*	Cumanda, Cumanda, Pastaza, East Ecuador	S 1.47686, W 78.14325
00N0836	*Turdus albicollis*	Estacion Cientifica Yasuni, Yasuni, Orellana, East Ecuador	S 0.67455, W 76.39837
07-0007	*Turdus albicollis*	Inselberg Station, Nouragues, French Guiana	N 4.09412, W 52.68438
07-0074	*Turdus albicollis*	Inselberg Station, Nouragues, French Guiana	N 4.09412, W 52.68438
07-0075	*Turdus albicollis*	Inselberg Station, Nouragues, French Guiana	N 4.09412, W 52.68438
07-0116	*Turdus albicollis*	Inselberg Station, Nouragues, French Guiana	N 4.09412, W 52.68438
07-0184	*Turdus albicollis*	Inselberg Station, Nouragues, French Guiana	N 4.09412, W 52.68438
07-0186	*Turdus albicollis*	Inselberg Station, Nouragues, French Guiana	N 4.09412, W 52.68438
07-0200	*Turdus albicollis*	Inselberg Station, Nouragues, French Guiana	N 4.09412, W 52.68438
07-0290	*Turdus albicollis*	Inselberg Station, Nouragues, French Guiana	N 4.09412, W 52.68438
07-0307	*Turdus albicollis*	Inselberg Station, Nouragues, French Guiana	N 4.09412, W 52.68438
07-0308	*Turdus albicollis*	Inselberg Station, Nouragues, French Guiana	N 4.09412, W 52.68438
99N5273	*Turdus albicollis*	Jatun Sacha Station, Misahualli, Napo, East Ecuador	S 1.0215, W 77.62144
00N3527	*Turdus albicollis*	Nueva Alianza, Sangay National Park, Morona-Santiago, East Ecuador	S 2.09883, W 78.15164
00N3530	*Turdus albicollis*	Nueva Alianza, Sangay National Park, Morona-Santiago, East Ecuador	S 2.09883, W 78.15164
08-0840	*Turdus albicollis*	Paracou Field Station, Paracou, French Guiana	N 5.27595, W 52.9280
08-0841	*Turdus albicollis*	Paracou Field Station, Paracou, French Guiana	N 5.27595, W 52.9280
08-0842	*Turdus albicollis*	Paracou Field Station, Paracou, French Guiana	N 5.27595, W 52.9280
08-0886	*Turdus albicollis*	Paracou Field Station, Paracou, French Guiana	N 5.27595, W 52.9280
08-0897	*Turdus albicollis*	Paracou Field Station, Paracou, French Guiana	N 5.27595, W 52.9280
08-0645	*Turdus albicollis*	Parare Station, Nouragues, French Guiana	N 4.04055, W 52.6757
08-0689	*Turdus albicollis*	Parare Station, Nouragues, French Guiana	N 4.04055, W 52.6757
08-0731	*Turdus albicollis*	Parare Station, Nouragues, French Guiana	N 4.04055, W 52.6757
00N0721	*Turdus albicollis*	Tiputini Biological Station, Tiputini, Orellana, East Ecuador	S 0.63698, W 76.14912

00N0748	*Turdus albicollis*	Tiputini Biological Station, Tiputini, Orellana, East Ecuador	S 0.63698, W 76.14912
00N5006	*Turdus albicollis*	Tiputini Biological Station, Tiputini, Orellana, East Ecuador	S 0.63698, W 76.14912
00N5073	*Turdus albicollis*	Tiputini Biological Station, Tiputini, Orellana, East Ecuador	S 0.63698, W 76.14912
04N6520	*Xenops minutus*	Bilsa Biological Station, Bilsa, Esmeraldas, West Ecuador	N 0.36076, W 79.71486
04N8453	*Xenops minutus*	Bilsa Biological Station, Bilsa, Esmeraldas, West Ecuador	N 0.36076, W 79.71486
04N8463	*Xenops minutus*	Bilsa Biological Station, Bilsa, Esmeraldas, West Ecuador	N 0.36076, W 79.71486
04N8539	*Xenops minutus*	Bilsa Biological Station, Bilsa, Esmeraldas, West Ecuador	N 0.36076, W 79.71486
05N7414	*Xenops minutus*	Bilsa Biological Station, Bilsa, Esmeraldas, West Ecuador	N 0.36076, W 79.71486
05N7418	*Xenops minutus*	Bilsa Biological Station, Bilsa, Esmeraldas, West Ecuador	N 0.36076, W 79.71486
05N8871	*Xenops minutus*	Bilsa Biological Station, Bilsa, Esmeraldas, West Ecuador	N 0.36076, W 79.71486
05N9023	*Xenops minutus*	Bilsa Biological Station, Bilsa, Esmeraldas, West Ecuador	N 0.36076, W 79.71486
05N9160	*Xenops minutus*	Bilsa Biological Station, Bilsa, Esmeraldas, West Ecuador	N 0.36076, W 79.71486
05N9645	*Xenops minutus*	Bilsa Biological Station, Bilsa, Esmeraldas, West Ecuador	N 0.36076, W 79.71486
05N9995	*Xenops minutus*	Bilsa Biological Station, Bilsa, Esmeraldas, West Ecuador	N 0.36076, W 79.71486
00N0890	*Xenops minutus*	Estacion Cientifica Yasuni, Yasuni, Orellana, East Ecuador	S 0.67455, W 76.39837
00N0554	*Xenops minutus*	Guagua Sumaco, Guagua Sumaco, Napo, East Ecuador	S 0.72493, W 77.57564
07-0042	*Xenops minutus*	Inselberg Station, Nouragues, French Guiana	N 4.09412, W 52.68438
08-0881	*Xenops minutus*	Paracou Field Station, Paracou, French Guiana	N 5.27595, W 52.9280
00N0676	*Xenops minutus*	Tiputini Biological Station, Tiputini, Orellana, East Ecuador	S 0.63698, W 76.14912
00N0747	*Xenops minutus*	Tiputini Biological Station, Tiputini, Orellana, East Ecuador	S 0.63698, W 76.14912
00N1053	*Xenops minutus*	Tiputini Biological Station, Tiputini, Orellana, East Ecuador	S 0.63698, W 76.14912
00N5019	*Xenops minutus*	Tiputini Biological Station, Tiputini, Orellana, East Ecuador	S 0.63698, W 76.14912
00N5069	*Xenops minutus*	Tiputini Biological Station, Tiputini, Orellana, East Ecuador	S 0.63698, W 76.14912
05N0057	*Xenops minutus*	Tiputini Biological Station, Tiputini, Orellana, East Ecuador	S 0.63698, W 76.14912
05N0066	*Xenops minutus*	Tiputini Biological Station, Tiputini, Orellana, East Ecuador	S 0.63698, W 76.14912
05N0077	*Xenops minutus*	Tiputini Biological Station, Tiputini, Orellana, East Ecuador	S 0.63698, W 76.14912
