# Supplementary material for: A Trans-Amazonian Screening of mtDNA Reveals Deep Intraspecific Divergence in Forest Birds and Suggests a Vast Underestimation of Species Diversity
Source: PLoS One. 2012 Jul 17;7(7):e40541. doi: 10.1371/journal.pone.0040541 (PMC3398903; doi:10.1371/journal.pone.0040541)
Supplement: Table S2 — Descriptions of phenotypic characteristics of eastern and western Amazonian populations of target species. (PDF) [file pone.0040541.s002.pdf]

Table S2. Phenotypic characteristics of eastern and western Amazonian populations of target species. Descriptions based on Restall et al. (2006). EC = Ecuador, FG = French Guiana.

Species	Subspecies EC	Subspecies FG	Phenotype EC	Phenotype FG	Phenotypic score
Geotrygon montana	*G. m. montana*	*G. m. montana*	No differences reported.	No differences reported.	1
Phaetornis bourcieri	*P. b. bourcieri*	*P. b. whitelyi*	Whitish throat, pale buffy flanks, more greenish crown, golden-buffy edges to uppertail-coverts.	Buffy throat and flanks; paler edges to uppertail-coverts	2
Campylopterus largipennis	*C. l. aequatorialis*	*C. l. largipennis*	White restricted to tip of rectrices.	White distal half of tail.	3
Thalurania furcata	*T. f. viridipectus*	*T. f. furcata*	Male: nape green; green gorget extends to breast; undertail-coverts fringed white.	Male: nape blue; gorget restricted to throat; undertail-coverts fringed pale blue.	4
Trogon rufus	*T. r. sulphureus*	*T. r. rufus*	Eye ring yellow in both sexes; male breast green; male tail reddish-coppery with green subterminal and black terminal bands.	Eye ring blue in both sexes; male breast cobalt-blue; male tail pale-olivaceous green with black terminal band.	4
Chloroceryle aenea			No differences reported.	No differences reported.	1
Momotus momota	*M. m. microstephanus*	*M. m. momota*	Nape violet-blue.	Nape rufous.	4
Galbula albirostris	*G. a. chalcocephala*	*G. a. albirostris*	Male: crown reddish-purple, chin partially dusky, underparts deep rufous.	Male: crown deep maroon, chin buff, underparts cinnamon.	3
Philydor erythrocercum	*P. e. subfulvum*	*P. e. erythrocercum*	Generally paler, with eyebrow and throat pale buffy, underparts washed more greyish-olive.	Generally more rufescent, more rufous on rump, eyebrow more orange.	2
Automolus ochrolaemus	*A. o. turdinus*	*A. o. turdinus*	No differences reported.	No differences reported.	1
Xenops minutus	*X. m. obsoletus*	*X. m. ruficaudus*	Whitish throat more extensive, and feathers have fine brownish fringes; some chest streaking.	Darker crown; narrow buff streaks and broader brown fringes to throat feathers; obvious chest streaking.	2
Sclerurus mexicanus	*S. m. peruvianus*	*S. m. macconelli*	Rump and upper tail coverts almost concolorous with back; orange only on throat.	Rump and uppertail-coverts bright rufous; orange-rufous of throat extends to breast and belly.	3
Dendrocincla fuliginosa	*D. f. neglecta* / *D. f. phaeochroa*	*D. f. fuliginosa*	Slightly darker and more olivaceous above; whiter throat; no streaks on throat; no postocular markings; legs and feet blue-grey.	Chin and throat buffy, flecked dusky and white, with pale shaft streaks; postocular row of tawny-orange spots; legs and feet black.	4
Glyphorynchus spirurus	*G. s. castelnaudii*	*G. s. spirurus*	Variable, throat cinnamon, generally darker, more olivaceous below; shorter, stouter bill.	Chin and throat pale orange, scaled dark olive, pale buffy spots on breast large and diamond-shaped, fine streaks on belly.	3
Thamnomanes ardesiacus	*T. a. ardesiacus*	*T. a. obidensis*	Male: black resticted to chin.	Male: full black chin and throat.	3
Thamnomanes caesius	*T. c. glaucus*	*T. c. glaucus*	No differences reported.	No differences reported.	1

Myrmotherula axillaris	*M. a. melanea*	*M. a. axillaris*	Male: much blacker above, charcoal rather than slate, and black more extensive on vent, wings and tail darker and undertail-coverts with a little black on centres; less white on flanks. Female: olive above, darker on sides, paler below.	Male: slate grey above with white outer fringes to scapulars; undertail-coverts subterminally black with white fringes. Female: rufescent olive above.	3
Myrmotherula longipennis	*M. l. zimmeri*	*M. l. longipennis*	Female: head darker greyish-olive, back bluish-grey, wings and tail darker with more contrasting cinnamon spots on wings; cinnamon below.	Female: olive-brown above with slight greyish tone, rufous-buffy fringes to wing-coverts and tips of tail; whitish belly.	4
Myrmotherula menetriesii	*M. m. pallida*	*M. m. cinereiventris*	Male: uniform grey. Female: grey above, tinged olive, underparts rich cinnamon.	Male: slightly darker, with occasional black dot on breast. Female: brownish-olive above, distinctly paler ochraceous below with slight grey wash on flanks.	4
Hypocnemis cantator	*H. c. saturata*	*H. c. cantator*	Yellow throat to breast, with a few deliberate black streaks on sides; brighter rufous below.	White chin to belly, with some streaking; flanks to undertail-coverts rich rufous.	4
Hylophylax naevius	*H. n. theresae*	*H. n. naevius*	More extensive black and more extensively streaked pale on back; more extensively streaked black below; larger white tips to tail.		2
Hylophylax poecilinotus	*H. p. lepidonotus*	*H. p. poecilinotus*	Female: entire underparts pale rufous, yellowish-ochre belly.	Female: top and sides of head deep rufous; chin tinged pale rufous; throat white; breast to undertail-coverts grey, tinged pale rufescent on flanks; thighs rufous.	4
Pithys albifrons	*P. a. peruvianus*	*P. a. albifrons*		Narrow white postocular line. [Note: Grey nape, as described in Restall et al. 2006, is an error]	4
Formicarius colma	*F. c. nigrifrons*	*F. c. colma*	Black usually to forecrown; legs and feet vinaceous	Black restricted to forehead; legs and feet dark vinaceous	2
Formicarius analis	*F. a. zamorae*	*F. a. crissalis*	Very dark with intense colours, very small white loral spot.	Palest race, bright pale chestnut patch behind ear-coverts and large white loral spot.	4
Conopophaga aurita	*C. a. occidentalis*	*C. a. aurita*	Male: darker above with heavy black scallops, rufous more extensive on breast. Female: white chin.	Fine black scallops on back and belly mainly clear white. Female: white chin and thoat.	3
Mionectes oleagineus / M. macconelli	*M. o. hauxwelli*	*M. m. macconelli*	Olive abow with two diffuse ochraceous wingbars.	Dark olive-green above, with no wingbars.	4
Corythopis torquatus	*C. t. sarayacuensis*	*C. t. anthoides*	Crown concolorous with brownish-olive back.	Slightly smaller; crown slate grey.	2
Rhynchocyclus olivaceus	*R. o. aequinoctialis*	*R. o. guianensis*	Yellow fringes to wing-coverts.	Yellow-ochre fringes to wing-coverts.	2
Platyrinchus coronatus	*P. c. coronatus*	*P. c. gumia*		Smaller, with brighter yellow belly.	2
Myiobius barbatus	*M. b. barbatus*	*M. b. barbatus*	No differences reported.	No differences reported.	1

Pipra erythrocephala	*P. e. berlepschi*	*P. e. erythrocephala*	Uniform yellow head with narrow, sharply defined red border.	Orange head with variable reddish suffusion.	3
Dixiphia pipra	*D. p. coracina*	*D. p. pipra*	Male: grey bases to white feathers. Female: clean grey top of head and nape, whitish-yellow head-sides, bright yellowish bellow.	Male: pure white cap. Female: olive-green above with greyish tone to head, grey underparts washed olive-green, slightly paler on belly and vent.	4
Schiffornis turdina	*S. t. aenea* (E slope of Andes) / *S. t. amazona* (NE lowlands)	*S. t. wallacii*	*S. t. aenea* has browner crown than *S. t. amazona* .	Like *amazona*	2
Hylophilus ochraceiceps	*H. o. ferrugineifrons*	*H. o. luteifrons*	Tawny forecrown; grey underparts washed narrowly olive on sides and flanks; eyes yellowish white.	Yellowish-rufous forecrown; throat greyish or yellowish-white, buffier below, specially on breast; eyes brown.	4
Cyphorhinus arada	*C. a. salvini*	*C. a. arada*	Lacks bold streaks on mantle; bright chestnut eyebrow starts at lores and supraloral, bright chestnut from chin to breast and sides; rest of underparts deep chestnut-brown but not as dark as upperparts; lacks barring on undertail coverts.	Nape and mantle streaked with long white "teardrops", postocular white eyebrow washed chestnut,lower breast and upper belly pale buffy, breast-sides, flanks and lower belly to undertail-coverts brown with black streaks on sides; barring on undertail coverts	4
Microcerculus bambla	*M. b. albigularis*	*M. b. bambla*	Similar to *bambla* , but evenly and finely barred from forehead to uppertail-coverts; very pale grey chin and throat; grey breast and sides	Slightly greyish-brown above with regular and even black bars on rump to uppertail-coverts, throat broadly mauve-grey.	4
Turdus albicollis	*T. a. spodiolaemus*	*T. a. phaeopygus*	More rufous -olive above and heavier throat streaking; lower mandible yellow; legs and feet dark horn.	Bill black, legs and feet grey.	4
Lanio fulvus	*L. f. peruvianus*	*L. f. fulvus*		Generally more fulvescent, with only nape bright yellow.	2
Tachyphonus surinamus	*T. s. brevipes*	*T. s. surinamis*	Male: buffy-white pectoral tuft . Female: paler below, creamy buff to pale cinnamon on undertail-coverts.	Male: crest longer with a few black tips to central feathers, pectoral tufts white. Female: pale cinnamon below with cinnamon undertail coverts; eye-ring more prominent.	2
Cyanocompsa cyanoides	*C. c. rothschildii*	*C. c. rothschildii*	No differences reported.	No differences reported.	1
